# Optimisation of an exemplar oculomotor model using multi-objective genetic algorithms executed on a GPU-CPU combination

**DOI:** 10.1186/s12918-017-0416-2

**Published:** 2017-03-24

**Authors:** Eleftherios Avramidis, Ozgur E. Akman

**Affiliations:** 1 Centre for Systems, Dynamics and Control, College of Engineering, Mathematics and Physical Sciences, University of Exeter, North Park Road, Exeter, EX4 4QF UK; 2 Department of Electronic Engineering, National University of Ireland, Maynooth, Ireland

**Keywords:** Systems biology, Parameter optimisation, Multi-objective genetic algorithms, High-performance computing, Oculomotor control, Mathematical modelling, Infantile nystagmus

## Abstract

**Background:**

Parameter optimisation is a critical step in the construction of computational biology models. In eye movement research, computational models are increasingly important to understanding the mechanistic basis of normal and abnormal behaviour. In this study, we considered an existing neurobiological model of fast eye movements (saccades), capable of generating realistic simulations of: (i) normal horizontal saccades; and (ii) infantile nystagmus – pathological ocular oscillations that can be subdivided into different waveform classes. By developing appropriate fitness functions, we optimised the model to existing experimental saccade and nystagmus data, using a well-established multi-objective genetic algorithm. This algorithm required the model to be numerically integrated for very large numbers of parameter combinations. To address this computational bottleneck, we implemented a master-slave parallelisation, in which the model integrations were distributed across the compute units of a GPU, under the control of a CPU.

**Results:**

While previous nystagmus fitting has been based on reproducing qualitative waveform characteristics, our optimisation protocol enabled us to perform the first direct fits of a model to experimental recordings. The fits to normal eye movements showed that although saccades of different amplitudes can be accurately simulated by individual parameter sets, a single set capable of fitting all amplitudes simultaneously cannot be determined. The fits to nystagmus oscillations systematically identified the parameter regimes in which the model can reproduce a number of canonical nystagmus waveforms to a high accuracy, whilst also identifying some waveforms that the model cannot simulate. Using a GPU to perform the model integrations yielded a speedup of around 20 compared to a high-end CPU.

**Conclusions:**

The results of both optimisation problems enabled us to quantify the predictive capacity of the model, suggesting specific modifications that could expand its repertoire of simulated behaviours. In addition, the optimal parameter distributions we obtained were consistent with previous computational studies that had proposed the saccadic braking signal to be the origin of the instability preceding the development of infantile nystagmus oscillations. Finally, the master-slave parallelisation method we developed to accelerate the optimisation process can be readily adapted to fit other highly parametrised computational biology models to experimental data.

**Electronic supplementary material:**

The online version of this article (doi:10.1186/s12918-017-0416-2) contains supplementary material, which is available to authorized users.

## Background

### Oculomotor control

In human eyes, visual acuity is highest in the foveal region of the retina, and only when the object of interest is held steady on this region. Consequently, the oculomotor system has evolved to perform eye movements that ensure these conditions are met. These eye movements can be voluntary or involuntary, and their role is to fixate the fovea to visual stimuli, to track moving stimuli and to compensate for body movements [1]. Depending on the visual stimuli and the viewing conditions, up to five of the following subsystems are involved in the execution of an eye movement: saccadic, smooth pursuit, vestibular, optokinetic and vergence. The subsystem of interest in this study is the saccadic system. This performs the rapid movements of the eyes (saccades) that transfer gaze from one target to another [2].

Saccades exhibit highly stereotyped behaviour which are commonly summarised using three key relationships between saccadic metrics. The first two relationships state that the saccade duration increases nearly linearly with amplitude and that peak saccadic velocity increases nonlinearly with amplitude at a decreasing rate. These relationships are called the main sequence and have been found to hold for a wide range of normal activities, such as reading, free scene viewing, visual search and walking [3]. The third relationship describes how the velocity profile shape changes with amplitude [4]. It has been shown that the length of the accelerating segment of the velocity profile is roughly consistent for all saccade amplitudes, whereas the length of the decelerating segment increases with amplitude. This causes the velocity profile of small saccades to be symmetrical, whereas those of larger saccades are skewed [4].

### Infantile nystagmus

Infantile nystagmus (IN) is an eye movement disorder characterised by involuntary, bilateral, conjugate oscillations of the eyes. The oscillations begin at birth, or shortly afterwards, and are usually restricted to the horizontal plane, although vertical and torsional movements (and combinations of these) have also been observed [5, 6]. Infantile nystagmus can be idiopathic, or associated with sensory defects (e.g. cataract and oculocutaneous albinism) [6, 7]. The estimated prevalence of all forms of nystagmus is 24 per 10000 individuals, of which IN accounts for 14 per 10000 [8]. Nystagmus has a significant impact on the quality of life, with many individuals registered as partially sighted or blind [9]. This makes the development of potential treatments for nystagmus patients an important goal of clinical motor disorder research.

The key characteristics of IN include: the oscillation plane, the waveform amplitude and period, the slow and fast phase, the foveation window and the baseline oscillation (see Additional file 1: Figure S1) [10, 11]. Typically, a slow eye movement (the slow phase) takes the eye away from the target, and a fast eye movement (the fast phase) returns it to the target. An important characteristic of the nystagmus oscillation is the foveation window: this is the cycle interval within which the velocity of the eyes is low enough (< 4 deg/s) for the patient to be able to see clearly [12]. The baseline oscillation is a low frequency sinusoidal oscillation, the amplitude of which is correlated with the nystagmus amplitude [10].

There have been at least 12 different IN waveforms identified in clinical studies [12] and the particular waveform type observed is influenced by factors including gaze angle, stress, attention and arousal [5, 6]. These waveforms can be divided into three groups: pendular, jerk and dual jerk, depending on the waveform shape (see Additional file 1: Figure S2 for schematics of these different groups). Although these waveforms may appear unrelated, it has been shown that they can be described as combinations of different template waveforms, such as sawtooth and pendular oscillations [13]. In pendular nystagmus, slow eye movements move the eye towards and away from the visual target. Jerk waveforms are divided into two categories: unidirectional and bidirectional. In unidirectional jerk, there is one fast eye movement in each period of the oscillation and this is always towards the target. In bidirectional jerk, there are two fast eye movements per period in alternating directions, both of which move the eye towards the target. Unidirectional jerk waveforms are further subdivided into two subcategories: jerk with saccadic foveation and jerk with slow eye movement (SEM) foveation. Jerk with saccadic foveation is subdivided into pure jerk and jerk with extended foveation. Jerk with SEM foveation is subdivided into pseudo-cycloid and pseudo-jerk (see Additional file 1: Figure S2).

### Computational models of the oculomotor system

Computational models have proved important in generating and testing hypotheses regarding the mechanisms underlying normal and pathological functioning of the oculomotor system [1, 14–19]. Models based on control theory [14–16] were highly successful in systematically examining the neural substrate underlying the different oculomotor subsystems [1, 17]. A key and influential achievement of this approach was the concept of a neural circuit to convert eye velocity-coded signals into position-coded signals (a neural integrator), derived theoretically from the necessity to hold gaze steady during head movements [20]. The existence of such a neural structure was subsequently verified experimentally [21]. A complementary approach based on nonlinear dynamics emerged towards the end of the 1990s, aiming to understand how the interactions of specific populations of oculomotor neurons could generate both normal and abnormal eye movements. An example of this approach was the model of the saccadic system proposed by Broomhead et al. [22], which was shown to be able to simulate both normal saccades and IN waveforms. A key implication of this model was that IN can be treated as a dynamic disease, in which there is no structural damage to the system and pathological behaviour is instead due to the system’s parameters operating outside of their normal range [23].

### Optimising the parameters of computational biology models

Parameter optimisation (or model fitting) is the process of finding the particular combinations of a model’s parameter values that enable it to reproduce the experimentally measured behaviour of the system of interest in an automated fashion. It is a critical step in the construction and analysis of biological models [24], because determining the optimal parameter values enables alternative models to be systematically ranked and experimentally testable predictions to be formulated [24–30]. This allows the assumptions made in constructing a given model to be assessed, and also provides insights into possible modifications to the model to improve the accuracy of its predictions [24, 27–29].

The development of robust, computationally efficient parameter optimisation methods is currently a very active topic of research [31–35]. Indeed, optimisation methods have been used previously to fit the parameters of oculomotor models to experimental data. Cullen et al. [25] were one of the first to employ such methods, using them to build a mathematical model of the neurons that generate saccades. Their methods allowed them to investigate: (i) the dynamic latency of a saccadic neuron; (ii) the prediction capacity of a given model; (iii) whether a more complex model increased the prediction capacity of the model; and (iv) the relationship between the initial conditions and saccade trajectories. Pasquariello et al. [10] used parameter optimisation to fit a model to experimental recordings of the infantile nystagmus baseline oscillation. Their results showed that, on average, the amplitude of the baseline oscillation was half that of the corresponding nystagmus oscillation.

### The genetic algorithm

A widely used optimisation method for computational biology problems is the genetic algorithm (GA) [30]. The GA is a stochastic search method based on mimicking natural selection, in which an evolving population of candidate problem solutions (i.e. parameter sets) is used to find the optimal solution (the parameter set yielding the best fit to data) [36]. A fitness function (also referred to as an objective function) is applied to each solution, yielding a value that represents how successful it is in solving the problem (the goodness-of-fit). Based on the values of the fitness function, the GA applies a number of stochastic genetic operators to the population so as to guide the search towards the optimal solution. A major advantage of GAs over deterministic optimisation methods (e.g. gradient descent) is that they do not require any prior assumptions about the objective function to hold, such as continuity or smoothness, with respect to the model’s parameters.

These advantages notwithstanding, when the optimisation problem involves large sets of highly parametrised nonlinear equations being fitted to multiple datasets, evaluating the fitness function for each parameter combination in the population can be very computationally intensive. This effect is amplified by two further factors. Firstly, the stochastic nature of the GA means that multiple runs are necessary to check that the solutions have stably converged [36]. Secondly, the GA itself has a number of parameters which require tuning, including those specifying the fitness function. It is therefore necessary to systematically test different combinations of GA parameters to ensure that true optima are returned. Consequently, considerable efforts have been made to reduce the execution time of GAs. For a given set of GA parameters, the same fitness function and genetic operators are applied to the solutions in the population. This makes GAs very suitable to parallelisation and many parallel architectures have been developed to exploit this feature to achieve acceleration [36].

However, for problems involving realistic parameter numbers, high-performance computing (HPC) clusters (i.e. multiple connected computers) are required to obtain results within a reasonable timeframe. Although HPC clusters provide significant processing power, and the intrinsic parallel architecture of GAs allows them to be executed over the multiple central processing units (CPUs) within a cluster, they also have a number of disadvantages. Chief amongst these are their purchase price, as well as the high costs associated with maintenance and energy consumption. An alternative approach is to use consumer-level graphics processing units (GPUs). These possess parallel computing power that can be comparable to that of the CPUs in a small HPC cluster. Furthermore, GPUs have a considerably lower cost and require substantially less maintenance. Recently, parallel GAs have been developed that run on GPUs, yielding significant speedups compared with CPUs [37–39].

### Multi-objective optimisation

When optimising a computational biology model, it can be the case that multiple, conflicting objectives have to be fitted simultaneously (e.g. datasets from different experimental protocols). This gives rise to a multi-objective optimisation (MOO) problem [40, 41], which can be expressed in general form as follows:

1 $$ \begin{array}{lll} \text{minimise} & f_{i}(\mathbf{x}), & i=1,\ldots,m, \\ \text{subject to:} & g_{j}(\mathbf{x})\leq{\mathit{b_{j}}}, & j=1,\ldots,k; \\ & g_{j}(\mathbf{x})=b_{j}, & j=k+1,\ldots,l. \end{array}   $$

Here, **x**=(*x*_1_,…,*x*_*n*_) is a vector which gathers together the parameters (decision variables) of the model and $\left \{f_{i} : \mathbb {R}\rightarrow \mathbb {R}, \, 1\le i \le m\right \}$ are the objective functions which quantify the quality of the model fit to different experimental features for each choice of **x**. Finally, the functions $\left \{g_{j}:\mathbb {R}\rightarrow \mathbb {R},\, 1\le j \le l\right \}$ impose any relevant constraints on the problem (e.g. that the parameters are within a biologically feasible range) and {*b*_1_,…,*b*_*l*_} are the corresponding constraint values [41–43]. Optimal solutions of (1) thus correspond to parameter sets that give the best fits to the experimental data, within the bounds specified.

One approach to solving such problems is the weighted-sum method [44], in which the objective functions *f*_*i*_(**x**) in (1) are summed with appropriate weights *w*_*i*_ to give a scalar objective function $\sum _{i} w_{i} f_{i}(\mathbf {x})$ to be minimised (uni-objective optimisation). However, this requires the *w*_*i*_ values that maximally exploit the information provided by the different objectives to be specified and these are typically not known a priori [41, 42, 45]. An alternative approach is the *ε*-constraint method, in which the MOO problem is reduced to a uni-objective problem by minimising one of the objectives and setting the others to be constrained to specific values [40, 46]. Different constraint values for each objective have to be explored in order to obtain solutions that provide a desirable trade-off between the objectives.

Other MOO methods are based on the concept of Pareto dominance, where a solution **x** is said to dominate a solution *x* ^′^ if and only if the following two conditions hold: first, solution **x** is no worse than *x* ^′^ on all objectives (*f*_*i*_(**x**) ≤ *f*_*i*_(**x** ^′^) for all *i*) and second, solution **x** is better on at least one objective (*f*_*j*_(**x**) < *f*_*j*_(**x** ^′^) for some *j*) [41, 42, 45, 47, 48]. The algorithms return an estimate of the set of solutions for which none of the objectives can be improved without compromising one of the others (the set of non-dominated solutions). These Pareto optimal solutions (the Pareto set) represent the optimal trade-off between the individual objectives. The image of this set in objective space is referred to as the Pareto front. An effective multi-objective optimisation algorithm returns an estimate of the Pareto set for which the corresponding front is close to the true one and on which the individual points are well separated in objective space [41, 42, 45, 48]. A good spread of solutions facilitates the selection of a final solution from the Pareto set. The method used for this final selection procedure is generally dependent on the particular problem of interest and often utilises the values of the Pareto optimal solutions on all objectives [49, 50].

MOO methods have been used previously in a range of bioinformatics and computational biology applications, such as RNA inverse folding [51], analysis and design of gene regulatory networks [52–54], and neural model fitting [55] (see [56] for a comprehensive review). A key advantage of MOO methods is that the structure of the Pareto set provides information about the conflicts between the objectives and the distribution of the possible solutions, which can yield useful insights into the particular biological system being modelled [56]. Among the most widely used MOO methods are multi-objective genetic algorithms (MOGAs). A very popular MOGA is the Non-dominated Sorting Genetic Algorithm II (NSGA-II) [45, 57]. The main characteristics of NSGA-II are elitism, a fast non-dominated sorting approach and a selection operator that ensures better spread of the solutions and better convergence near the Pareto front. NSGA-II has been successfully applied to a broad range of optimisation problems in different application areas [58–60].

### Aims and overview

The first aim of this computational study was to optimise the saccadic model of Broomhead et al. to existing experimental recordings of two types of eye movements: (i) infantile nystagmus oscillations; and (ii) horizontal saccades recorded from healthy subjects. Both optimisation problems are multi-objective, as conflicting objectives must be optimised together to reproduce key experimental characteristics. In the case of saccades, we optimised the model to experimental data measured for three different saccade amplitudes, treating each dataset as a separate objective. For nystagmus, we used the shape and period of each waveform as our objectives. We developed novel multi-objective fitness functions for each optimisation problem, and used NSGA-II to find Pareto optimal solutions in each case. Synthetic datasets generated from the model were used to help tune the NSGA-II parameters. The optimal solutions obtained in this fashion enabled us to quantify which experimental behaviours could be replicated by the model, thereby identifying possible modifications to increase the model’s predictive capacity.

The second aim of the study was to accelerate NSGA-II by parallelising its most computationally intensive component – the numerical integration of the oculomotor model – and executing the algorithm on a CPU-GPU combination. We show that for the optimisation of the oculomotor model (and any similar computational biology model), this approach can yield an order of magnitude decrease in computation time. Furthermore, we conclude that the selection of other NSGA-II processes for parallelisation in order to further improve performance will be dependent on the population size used.

## Methods

### The oculomotor model

The nonlinear dynamics model of the saccadic system proposed by Broomhead et al. [22] comprises the six coupled ordinary differential equations (ODEs) below:

2 $$\begin{array}{*{20}l} &\frac{dg}{dt}=v, \end{array} $$

3 $$\begin{array}{*{20}l} &\frac{dv}{dt}=\,-\,\left(\!\frac{1}{T_{1}}-\frac{1}{T_{2}}\right)v-\frac{1}{T_{1}T_{2}}g+\frac{1}{T_{1}T_{2}}n \\ & +\left(\frac{1}{T_{1}}+\frac{1}{T_{2}}\right)(r-l), \end{array} $$

4 $$\begin{array}{*{20}l} &\frac{dn}{dt}=-\frac{1}{T_{N}}n+(r-l), \end{array} $$

5 $$\begin{array}{*{20}l} &\frac{dr}{dt}=\frac{1}{\epsilon}\left(-r-\gamma rl^{2}+F(m)\right), \end{array} $$

6 $$\begin{array}{*{20}l} &\frac{dl}{dt}=\frac{1}{\epsilon}\left(-l-\gamma lr^{2}+F(-m)\right), \end{array} $$

7 $$\begin{array}{*{20}l} &\frac{dm}{dt}=-(r-l). \end{array} $$

The model was constructed on the basis of neurophysiological studies showing that the neural signal controlling saccades is generated by excitatory burst neurons (EBNs) in the brainstem [61]. This velocity-coded control signal (the pulse) is converted into a position-coded signal (the step) by the neural integrator. The pulse and step are then relayed to the extraocular muscles (the muscle plant), which move the eye to the desired position. Eqs. (2) and (3) model this muscle plant as a second order, overdamped linear system with time constants *T*_1_=0.15s and *T*_2_=0.012s [15, 62]; *g* and *v* represent the horizontal eye position (gaze angle) and eye velocity, respectively. Eq. (4) models the neural integrator signal *n*; this is characterised by the long time constant *T*_*N*_=25s, which is necessary to hold the eye steady at the target position following the saccade [62].

Equations (5) and (6) model the activities of the EBNs, which can be split into right (*r*) and left (*l*) populations, depending on the direction of motion that elicits maximal firing. The two neural populations suppress each other’s activity and this mutual inhibition is modelled by the terms *γ* *rl* ^2^ and *γ* *lr* ^2^ in Eqs. (5) and (6), respectively, with the parameter *γ* quantifying the inhibition strength. The saccadic velocity signal (the pulse) is the difference between the activities of the two EBN populations, *r*−*l*. For both populations, the EBN firing rate is a saturating nonlinear function of the motor error *m*, where *m* is the difference between the required and current eye positions. In the model, this function has the form:

8 $$ F(m)=\left\{ \begin{array}{lcc} \alpha^{\prime }\left(1-e^{-m/\beta^{\prime }}\right) & \text{if} & m\geq 0; \\ -\frac{\alpha }{\beta }me^{m/\beta} & \text{if} & m<0, \end{array} \right.  $$

which was derived from direct fits to experimental data (single-cell recordings from alert primates) [61].

The parameters *α* ^′^ and *β* ^′^ in (8) quantify the neurons’ response to error signals generated by motion in the direction of maximal firing (the on-response). *α* and *β* quantify the response to motion in the opposite direction (the off-response); the off-response causes a slowing of the eye towards the end of the saccade to prevent overshoot of the target and is referred to as the braking signal [61]. The parameter *ε* determines the speed at which the EBNs respond to the error signal, with smaller *ε* values yielding faster responses. Finally, (7) is the equation for the motor error. This was derived by assuming that *m*=*Δ* *g*−*s*, where *Δ* *g*=*T*−*g* is the eye displacement necessary to attain the target position *T*, and *s* is an estimate of eye displacement generated by a resettable integrator – separate to the NI – which also integrates the saccadic velocity command *r*−*l* [22].

In [22], Broomhead et al. empirically selected the values of the parameters *α* ^′^, *β* ^′^ and *γ* to match the saccadic main sequence and then examined the effect of varying *α* (off-response magnitude), *β* (off-response range) and *ε* (burst neuron response time). This manual parameter search revealed that the model is capable of generating accurate and inaccurate saccades, as well as several infantile nystagmus oscillations (see Additional file 1: Figure S3).

### Data

We used two types of data for the model optimisation: experimental and synthetic. The experimental data was taken from previous studies, and comprised a collection of nystagmus oscillations recorded during viewing at rest, together with mean saccadic velocity profiles recorded for different saccade amplitudes. The synthetic data was generated *de novo* from the saccadic model itself. Prior to optimising the model to experimental data, we performed fits to synthetic timeseries to assess: (i) how effective the multi-objective fitness functions were in reproducing experimental eye movement characteristics; and (ii) how the convergence and accuracy of the optimisation procedure varied with the NSGA-II parameters, thereby facilitating the final selection of these parameters. In particular, we checked that NSGA-II was consistently and reliably converging to the model parameters used to generate each synthetic dataset.

**Fig. 1:**
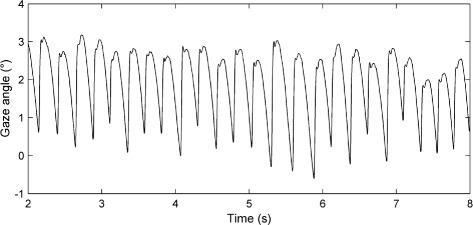
Segment of an experimentally recorded nystagmus time series. The vertical axis represents the horizontal gaze angle in degrees (°), with positive values denoting rightward eye positions. Time is in seconds (s)

**Nystagmus oscillations.** The experimental nystagmus database from which the data used in this study was taken consisted of horizontal eye movement recordings from 48 nystagmus patients enrolled on a drug trial [63]. From this database, data from 20 idiopathic subjects was chosen. The remaining 28 subjects in the drug trial had additional medical conditions and hence their data was rejected. The idiopath group consisted of 13 males and 7 females, with a mean age of 40.05±8.29. The eye movement recordings we used were those obtained before any drugs were administered to the participants. Binocular eye movements were recorded with the head stabilised by a chin rest. The sampling rate of the recordings was 250 Hz, with a resolution of 0.005 degrees. Typically, subjects had irregular waveforms with 4-6 cycles per second. An example of one of the nystagmus time series from the database is shown in Fig. 1.

The model parameters used to produce the synthetic IN waveforms are given in Additional file 1: Table S1; the waveforms themselves are shown in Additional file 1: Figure S4. These particular oscillations were chosen as fitting targets because they represent a broad distribution of amplitudes and periods.

**Fig. 2:**
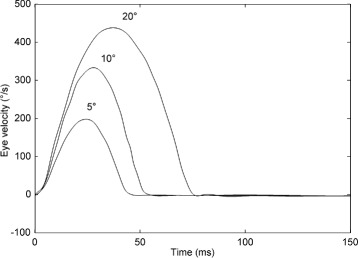
Experimentally recorded horizontal saccadic velocity profiles. Velocity profiles are shown for saccades of amplitude 5, 10 and 20 degrees. Velocity is in degrees per second (°/s); time is in milliseconds (ms). Data extracted from Fig. 2 of [64]

**Saccadic velocity profiles.** Experimental saccade data was taken from the study of Collewijn et al. [64]. The model was fitted to the mean velocity profiles reported in [64] for horizontal saccades of 5, 10 and 20 degrees (see Fig. 2). These particular saccade amplitudes were chosen because they are in the range of most frequently generated saccades [65, 66].

Synthetic saccadic velocity profiles were generated by the model using the parameter values listed in Additional file 1: Table S2; the velocity profiles themselves are shown in Additional file 1: Figure S5.

### The optimisation protocol

The goal of the optimisation protocol we developed was to find values of the six parameters { *α*,*β*,*ε*,*γ*,*α* ^′^,*β* ^′^} of the oculomotor model (2)-(7) that enabled it to reproduce the experimental eye movement recordings of interest. In the case of saccades, the model was fitted directly to experimental saccadic velocity profiles for each saccade amplitude. For nystagmus data, the model fitting involved finding simulated waveforms with the same amplitude, period and shape as the experimental data. For both optimisation problems, we employed the MOGA NSGA-II [57], using the implementation of the algorithm included in the MATLAB Global Optimisation Toolbox [67]. A key reason for our choice of optimiser was that NSGA-II has been successfully applied to optimisation problems with complex objective spaces [58–60]. Akman et al. [18] showed that the oculomotor model (2)-(7) has a rich bifurcation structure, incorporating Hopf, homoclinic, saddlenode, pitchfork and gluing bifurcations [68]. These bifurcations can create sharp changes in waveform type under small parameter variations, significantly compromising the ability of a standard optimiser to efficiently explore the parameter space.

### Formulating the fitness functions and selecting the NSGA-II parameters

In order to fit the model to the synthetic and experimental datasets, a fitness function for each data type (nystagmus oscillations and saccades) had to be formulated that measured the goodness-of-fit. When using a GA as an optimiser, different candidate fitness functions should be tested in order to assess the extent to which they are able to successfully quantify the target data characteristics [69]. The formulation of the fitness functions was a trial-and-error method. Each time we created a new fitness function (or modified an existing one), we tested it on the synthetic and experimental datasets to assess the effect of the change on the fitness function landscape and the quality of solutions obtained. For the nystagmus data, we initially used the squared difference between simulated and target waveforms. However, we found that a bi-objective fitness function based on oscillation period and shape gave better results. This observation is consistent with studies showing that multi-objective optimisers are less likely than uni-objective optimisers to become trapped in local minima [70, 71]. For the saccade data, we initially chose a multi-objective fitness function based on the saccadic main sequence. However, we found that this gave poor fits to the experimental data. We therefore formulated a multi-objective fitness function based on the squared difference between the experimental and simulated velocity profiles for different saccade amplitudes. The final versions of the fitness functions we used are described in greater detail in the “Fitness function for nystagmus waveforms” and “Fitness function for saccadic velocity profiles” sections below.

As mentioned previously, NSGA-II has parameters of its own (e.g. population size, mutation rate/type, crossover type, selection type) that affect the performance of the optimiser, as assessed by key benchmark measures such as the fitness value of the best solution and execution time. However, there are no general rules governing which choice of NSGA-II parameters is best for a given problem and so different combinations of these should also be tested [72]. Typically, given the stochastic nature of the GA, the goal is to find combinations of the GA parameters that allow it to converge stably to minima of the optimisation problem over multiple GA runs [36].

To quantify the accuracy and convergence properties of the optimal solutions found by NSGA-II, we used a hypervolume indicator. This was defined in terms of the volume $\mathcal {H\left (\hat {\mathcal {F}},\mathbf {y}_{R}\right)}$ in objective space formed between the estimated Pareto front $\hat {\mathcal {F}}$ and a fixed reference point **y**_*R*_ that was chosen to be dominated by all the points in $\hat {\mathcal {F}}$ [73]. To calculate an accurate approximation to this area we used the MATLAB function convhull.m. We compared this quantity to the volume $\mathcal {H}\left (\mathbf {0},\mathbf {y}_{R}\right)$ of the hypercuboid formed between **y**_*R*_, the origin **0** of the objective space and the points obtained by projecting **y**_*R*_ onto each objective axis separately. The ratio $\mathcal {H}\left (\hat {\mathcal {F}},\mathbf {y}_{R}\right)/\mathcal {H}\left (\mathbf {0},\mathbf {y}_{R}\right)$ of these two quantities provided a means for assessing whether the Pareto front estimates for a specific choice of fitness function and NSGA-II parameters were converging with generation number. Due to the stochastic nature of the optimiser, for each such choice, we calculated the mean and variance of this ratio over 16 independent optimisation runs, fixing the reference point **y**_*R*_ to be one dominated by all non-dominated sets produced collectively over the individual runs. In addition, to provide a measure that was more convenient for the visualisation of results, we subtracted the ratio from 1. The resulting measure $\mathcal {H}_{I}=1-\mathcal {H}\left (\hat {\mathcal {F}},\mathbf {y}_{R}\right)/\mathcal {H}\left (\mathbf {0},\mathbf {y}_{R}\right)$ was taken as the overall goodness-of-fit, with a value of 0 indicating a Pareto front comprised solely of the origin: i.e. a solution that perfectly fits all objectives simultaneously. The accuracy and convergence of the results were further evaluated by assessing how the minimum Euclidean distance $d_{\hat {\mathcal {F}}}=\min \left \{||\mathbf {y}||_{2}:\mathbf {y}\in \hat {\mathcal {F}}\right \}$ between the estimated Pareto front and the origin of objective space varied with population size (a minimum distance close to 0 indicates a good fit).

Each individual in the NSGA-II population encodes in floating point values a particular combination of the model parameters { *α*,*β*,*ε*,*γ*,*α* ^′^,*β* ^′^}. For each candidate fitness function and NSGA-II parameter combination, the initial population was uniformly distributed in the following parameter space:

9 $$  \begin{array}{lllllll} 1&\!\leq\alpha&\!\!\leq1000;\!\! \quad 0.1&\!\leq \beta &\!\!\leq 60;\!\! \quad 0.00001 &\!\leq \epsilon &\!\!\leq 0.1;\\ 0&\!\leq\gamma&\!\!\leq12;\!\! \qquad\,\, 50&\!\leq\alpha^{\prime}&\!\!\leq 1000;\!\! \qquad 0.1&\!\leq\beta^{\prime}&\!\!\leq 60. \end{array}  $$

The bounds on *α*,*β*,*α* ^′^ and *β* ^′^ were chosen based on the experimental findings of Van Gisbergen et al. [61], whereas the bounds on *ε* were based on the previous bifurcation analysis of the model [18]. The bounds on *γ* were derived from multiple preliminary NSGA-II runs which showed that the corresponding *γ* range returned solutions yielding biologically feasible waveforms (i.e. waveforms with shapes, periods and amplitudes consistent with experimental measurements). For each individual, the initial conditions of the model were set to

10 $$\begin{array}{*{20}l} g(0)=v(0)=n(0)=r(0)=l(0)=0; \quad m(0)=\Delta g, \end{array} $$

simulating a saccade of amplitude *Δ* *g* initiated from rest in the primary position (0 degrees – looking directly ahead). For nystagmus fitting, *Δ* *g* was set to 1.5, simulating a rightward saccade of 1.5 degrees, while for saccade fitting, *Δ* *g* was set to each of the different saccade amplitudes (5, 10 and 20 degrees) in turn.

The NSGA-II parameters that we varied to explore optimisation performance were as follows: the population size (values tested were 500, 1000, 2000, 4000 and 8000); the crossover function (intermediate or heuristic) and the distance measure type (phenotype or genotype). We did not vary all the NSGA-II parameters as this would have increased the computation time considerably, making the process impractical. Accordingly, in order to keep the running time short, the number of generations was fixed at 100 for saccade and synthetic nystagmus fits and at 200 for experimental nystagmus fits. The selection type was set to binary tournament (this selects each parent by randomly drawing two individuals from the population and then chooses the one with the highest fitness for crossover), and the mutation type was set to adaptive feasible (this mutates individuals towards regions in parameter space which – based on previous generations – are more likely to give better fitness).

It was expected that the population size would be a key determinant of NSGA-II accuracy and convergence, since if the population size is too small, the algorithm is unable to converge to the Pareto front, whilst increasing the population size requires greater computational resources [74]. Hence, when choosing the population size, a balance needs to be found between accuracy/convergence and the resulting computational load (the significant increase in computation time with population size can be seen for synthetic nystagmus data in Additional file 1: Table S3). Final population sizes were chosen to provide a reasonable trade-off between accuracy/convergence and running time. In the case of the crossover method, we found that heuristic crossover returned good results for considerably smaller population sizes compared with intermediate crossover. Of the remaining NSGA-II parameters, best results were obtained with the phenotype-based distance measure.

### Fitness function for nystagmus waveforms

For nystagmus waveforms, the multi-objective fitness function evaluated each individual to extract two objectives: (i) the difference in shape between the target and the scaled-to-target-period simulated waveforms; and (ii) the difference in period between the waveforms. An amplitude comparison was not included, as exploratory NSGA-II runs on both synthetic and experimental waveforms indicated that it was redundant: the shape comparison was sufficient for the optimiser to yield solutions with the correct amplitude. The input to the fitness function was a single period of the target waveform and a single period of the simulated time series.

**Extracting a single period of a simulated waveform.** The procedure for extracting a single period of a simulated oscillation – either for use as a target waveform or for fitting – was as follows. First, the model was integrated for 6 s. This time interval was chosen because exploratory runs of the method showed that it enabled one period of the waveform to be reliably extracted across a broad range of parameter values. The initial 2.4 s of the time series was then discarded as transient dynamics preceding the onset of oscillations. Next, the remaining portion of the time series was normalised to lie in the interval [0,1] and the local minima of the oscillation were identified. A single period of the waveform was then obtained by extracting all the points with value below 0.2 and between the last two local minima in the time series. If no such points were found, then the input was classified as non-oscillatory and the process terminated, with the individual assigned an arbitrarily high value (10^60^) for each objective.

**Extracting a single period of an experimental nystagmus recording.** For experimentally recorded waveforms, the extraction of a single period was much more challenging due to the nondeterministic nature of real nystagmus oscillations, in which each successive cycle has a slightly different period, amplitude and shape (see Fig. 1). This raises the question of which cycle(s) should be chosen for the fitting procedure. To address this, we used a variant of the nonlinear time series analysis method developed by So et al. [75, 76]. Starting from a scalar sequence of system measurements, this allows one to find either unstable periodic orbits (UPOs) generated by a deterministic system, or stable periodic orbits which have been destabilised by a noise process [75–79]. The method is based on the assumption that there is a significant deterministic component to the experimental system of interest, and so the extracted periodic orbits can be used to characterise the system’s dynamics by providing single oscillation cycles that are representative of the observed nonperiodic waveforms [23, 77]. This method has been previously applied successfully to nystagmus recordings [78, 79]. Here, we used the version of the method developed by Theodorou et al. [79], which we describe below.

The first stage of the method involves identifying the intervals { *τ*_1_,*τ*_2_,…,*τ*_*N*_} between individual nystagmus cycles by thresholding the velocity time series. The velocity threshold was chosen to correspond roughly to the middle of the fast phase of each nystagmus waveform. Next, we applied the method of delays [80, 81] to the interval data. In the method of delays, a sliding window of *d* samples is moved through the data, generating a sequence of *d*-dimensional vectors. Given the interval time series {*τ*_1_,*τ*_2_,…,*τ*_*N*_}, the delay vectors {**w**_1_,**w**_2_,…,**w**_*N*−*d*+1_} are defined as **w**_*k*_=(*τ*_*k*_,*τ*_*k*+1_,…,*τ*_*k*+*d*−1_)^*T*^. The evolution of the delay vectors is governed by a nonlinear map **F**, via **w**_*k*+1_=**F**(**w**_*k*_), which – under certain genericity conditions – is related to the corresponding map governing the evolution of inter-cycle intervals in the underlying experimental system by a smooth, invertible change of coordinates [80, 81]. This means that fixed points of **F**, which are points in the delay space **w**_∗_ for which **F**(**w**_∗_)=**w**_∗_, correspond to periodic cycles in the ambient system. For such points, *τ*_1_=*τ*_2_=⋯=*τ*_*N*_=*τ*_∗_; fixed points therefore lie on the long diagonal of the delay space (the set of *d*-dimensional vectors with equal entries). Candidate fixed points can therefore be identified by looking for delay vectors that lie close to this diagonal.

The method of So et al. [75, 76] facilitates this identification step by applying a data transformation **G** to the delay vectors that concentrates them onto the fixed points. This data transformation is given by:

11 $$ \mathbf{G}(\mathbf{w}_{n})=\left(I-D\mathbf{F}(\mathbf{w}_{n})\right)^{-1}(\mathbf{w}_{n+1}-D\mathbf{F}(\mathbf{w}_{n})\mathbf{w}_{n}).   $$

In Eq. (11), *I* is the *d*×*d* identity matrix and *D* **F**(**w**_*n*_) is the *d*×*d* Jacobian matrix of **F** evaluated at **w**_*n*_, which is estimated from the delay vectors using finite differences [79]. The transformed data lying within a small cross-sectional tube along the delay space diagonal is then projected onto the diagonal itself. Fixed points are identified as sharp peaks in the histogram obtained by binning the 1-dimensional projected data [76].

To extract UPOs from the nystagmus recordings using this method, we chose the embedding dimension *d* to be 2, the diameter of the cross-sectional tube to be 0.5 units and the histogram bin size to be 0.025 s [78]. Candidate period orbits were then identified as those with inter-cycle intervals *τ*_*k*_ lying within 0.0125 s of the histogram peak *τ*_∗_. From this collection of periodic waveforms, we choose the cycle with the smallest difference between its first and last points. Because it was more convenient to use waveform targets that start at the beginning of the fast phase, we concatenated three copies of the waveform and chose the section between two successive fast phases as the final cycle to be fitted. An example of the application of this procedure to an experimental nystagmus waveform is shown in Additional file 1: Figure S6.

**Calculating the shape and period difference.** In order to calculate the shape difference, the extracted waveform was scaled to the target waveform in time so that they had the same period. Subsequently, cubic interpolation was applied to the scaled waveform to give it the same time mesh as the target. The difference in shape *d*_*S*_ was then calculated as the sum-of-squares

12 $$ d_{S} = \sqrt{\frac{1}{N}\sum_{i=1}^{N} \left(S_{i}-T_{i}\right)^{2}},  $$

where *S*_*i*_ and *T*_*i*_ denote the values of the scaled and target waveforms, respectively, at time *t*_*i*_, where *t*_*i*+1_−*t*_*i*_ is the (fixed) data sampling interval.

The period difference *d*_*p*_ was calculated as:

13 $$ d_{P} = \left| \tau_{E}-\tau_{T} \right|,  $$

where *τ*_*E*_ is the period of the extracted, unscaled waveform and *τ*_*T*_ is the period of the target waveform.

### Fitness function for saccadic velocity profiles

For saccades, the input to the fitness function was the experimental velocity profiles for amplitudes of 5, 10 and 20 degrees and the corresponding simulated profiles generated by the model. The fitness function therefore comprised three objectives {*d*_1_,*d*_2_,*d*_3_}, defined as the sum-of-squares difference between the simulated and target data for each saccade amplitude:

14 $$ d_{j} = \sqrt{\frac{1}{N}\sum_{i=1}^{N} \left(S_{ij}-T_{ij}\right)^{2}}.  $$

Here, *S*_*ij*_ and *T*_*ij*_ denote the values of the simulated and target profiles, respectively, at time *t*_*i*_, where *t*_*i*+1_−*t*_*i*_ is the (fixed) data sampling interval and *j* indexes the amplitude. To extract the simulated velocity profile that was compared to the target one, we set the simulated velocity profile’s end point to that of the target profile, thereby ensuring that both had the same length.

### Selection of the best individual

When NSGA-II terminates, it returns a set of points that approximates the Pareto front. For nystagmus fitting, to obtain a single best-fit parameter set from this front, we selected the individual with the smallest difference in period to the target waveform, as this consistently provided a very good shape fit as well. We also explored the effect of selecting the individual yielding the lowest value on each objective separately (i.e. we chose the solutions from the Pareto set giving the best fits to saccades of 5, 10 and 20 degrees).

### Acceleration of the optimisation method using GPUs

In order to reduce computation time, we utilised the parallel capabilities of GPUs using a parallel implementation based on the master-slave model [36, 82]. In this implementation, for each instance of NSGA-II, the most computationally intensive task is distributed to multiple processors (the computing units of the GPU), while a master processor (the CPU) is used to control the parallelisation and run the remaining tasks.

In designing our optimisation protocol, we used the MATLAB profiler – which measures the execution time of each MATLAB function – to compare the time required to integrate the model to that required to perform the NSGA-II operations and evaluate the fitness function. Additional file 1: Figure S7 shows the mean execution time of each task, calculated from 8 independent NSGA-II instances run for 5 generations each with different population sizes. It can be seen that for all population sizes tested, the model integration was the most computationally intensive task. Hence, for our application, we chose to implement this process only on the GPU, with the genetic operations and fitness evaluation being performed by the CPU. Moreover, we employed the independent runs parallel model [36], in which multiple master-slave NSGA-IIs are executed independently on the CPU-GPU combination, with no communication between them.

**Fig. 3:**
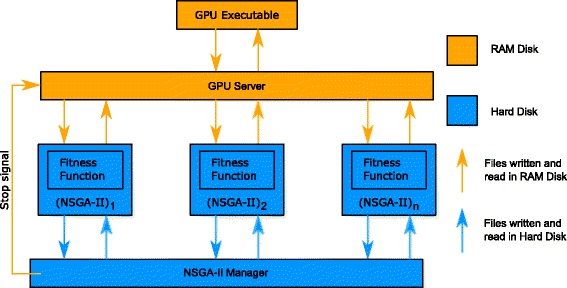
Flow diagram showing the method for running multiple NSGA-II instances in parallel. { (NSGA-II)_1_, (NSGA-II)_2_,..., (NSGA-II)_*n*_} are individual MATLAB NSGA-II instances created by the NSGA-II Manager. The GPU server is the interface between the NSGA-II instances and the GPU executable that performs the integration of the oculomotor model for each parameter set sent by the GA instances. *Arrows* indicate communication between different components using files: *orange arrows* indicate that the file is saved on the RAM disk, whereas *blue arrows* indicate that it is saved to the hard disk

**Running multiple NSGA-II instances in parallel.** To implement the parallelisation outlined above, we used the program organisation shown schematically in Fig. 3. The general idea behind the method is as follows: an NSGA-II manager program (written in MATLAB), which runs on the CPU, creates the individual NSGA-II MATLAB instances that run on the CPU in parallel. Each of these NSGA-II instances sends the parameter combinations (individuals) comprising its population to the GPU server program (another MATLAB instance), which writes these combinations to an input file. This file is read by the GPU executable (described in Additional file 1), which calculates the oculomotor model solutions. The results of the GPU executable are written to a binary file that is split by the GPU server program into multiple binary files containing the solutions corresponding to each NSGA-II instance. Finally, the NSGA-II instances read the binary files, evaluate the fitness of each individual in the population and then apply genetic operations to create the new population.

Running multiple instances of NSGA-II caused two main problems, due to the very large files that were generated. In particular, writing to (and reading from) the hard disk takes a considerable amount of time compared to writing to the RAM, thereby creating a speed bottleneck. To address this, we used a RAM drive – a block of the system RAM that the software uses as a disk drive – which proved to be considerably faster.

**Hardware used.** We tested our executable on four GPUs and one CPU. The GPU models were the AMD FirePRO W8100, the AMD Radeon HD 7970 GHz Edition, the NVIDIA Tesla K20m and the NVIDIA Tesla K40c. The CPU model was the Intel Core i7-4790K CPU. The reason we chose these particular GPUs is that they provide high double-precision compute capabilities which are required for the integration of stiff ODE systems. We chose to compare the performance of the above GPUs to the Intel Core i7-4790K, as it is one of the high-end CPU models currently available.

## Results

### Fits to synthetic nystagmus waveforms

Additional file 1: Figures S8-S11 show how the hypervolume indicator and minimum Pareto front distance vary with generation number when optimising the oculomotor model to each synthetic nystagmus waveform in turn. The results suggest that for each waveform, the Pareto front estimate is close to convergence by 100 generations for a population size of 4000. Indeed, increasing the population size further did not produce better results that would justify the extra computational resources required.

The boxplots in Additional file 1: Figures S12 and S13 show the optimised parameter values obtained for each population size, comparing them with the target parameter values used to generate the synthetic waveforms in each case. It can be seen that the optimised values for parameters *α*,*β*,*ε* and *γ* are fairly close to the target values for population sizes ≥4000, consistent with the hypervolume results. However, in the case of the parameters *α* ^′^ and *β* ^′^, there is no significant improvement in accuracy with increasing population size. Despite this, for a population size of 4000, the optimised waveforms are almost identical to the synthetic ones, confirming that the optimal NSGA-II parameters chosen in this case are sufficient to obtain a very good fit (see Additional file 1: Figure S14). For this population size, the optimised parameters are listed in Additional file 1: Table S1 and the corresponding coefficients of variation are plotted in Additional file 1: Figure S15.

These results confirm that applying NSGA-II to the shape-period nystagmus fitness function provides correct results when the parameter values of the model are already known.

### Fits to experimental nystagmus waveforms

**Fig. 4:**
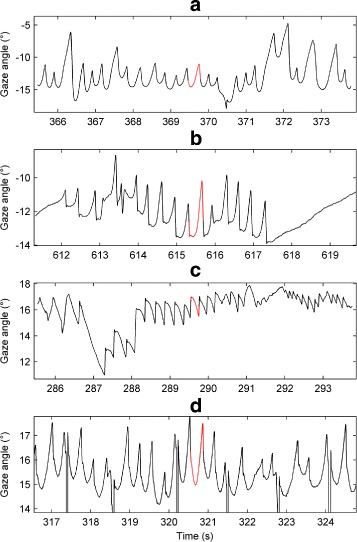
Segments of the experimental nystagmus time series used for model fitting. From *top* to *bottom*, the nystagmus waveform types are: asymmetric pseudo-cycloid (**a**); jerk with extended foveation (**b**); jerk (**c**); and asymmetric pseudo-cycloid (**d**). The extracted UPOs used for evaluating fitness are shown in *red*. On each plot, the vertical axis represents the horizontal gaze angle in degrees (°), with positive values denoting rightward eye positions. Time is in seconds (s). In the *bottom* plot (**d**), the *vertical lines* correspond to blinks

Four experimental time series from the nystagmus database were used for model fitting (see Fig. 4). These particular time series were selected as their morphologies resembled those generated by the model (i.e. jerk, jerk with extended foveation and asymmetric pseudo-cycloid). As discussed in Additional file 1, best results were obtained when the sampling frequency was 2500Hz. We therefore upsampled the extracted UPOs from 250Hz to 2500Hz by applying spline interpolation. Moreover, before being used as fitting targets, the extracted UPOs were normalised so that they started from the fast phase of the oscillation, with the fast phase in the rightward direction (see Additional file 1: Figure S16).

**Fig. 5:**
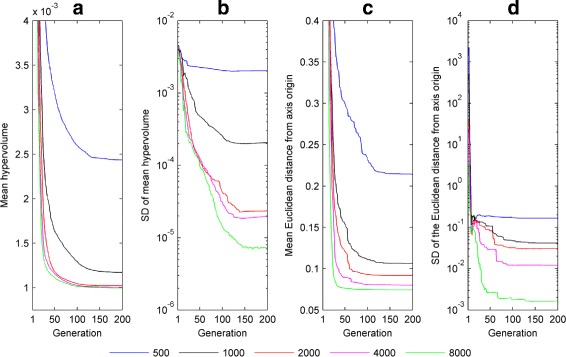
Convergence metrics of NSGA-II when fitting the model to experimental nystagmus waveform **a** of Fig. 4. **a** Mean value of the hypervolume indicator $\mathcal {H}_{I}$ as a function of generation number *n*. **b** Standard deviation (SD) of $\mathcal {H}_{I}$ as a function of *n*. **c** Mean value of the smallest Euclidean distance $d_{\hat {\mathcal {F}}}$ between the Pareto front estimate and objective space origin as a function of *n*. **d** SD of $d_{\hat {\mathcal {F}}}$ as a function of *n*. Convergence metrics were calculated from 16 runs of NSGA-II each for the following population sizes: 500, 1000, 2000, 4000 and 8000. The equivalent plots for waveforms **b**, **c** and **d** can be seen in Additional file 1: Figure S17, Additional file 1: Figure S18 and Additional file 1: Figure S19, respectively

Figure 5 shows how the hypervolume indicator and minimum Pareto front distance vary with generation number when fitting the model to one of the experimental asymmetric pseudo-cycloid waveforms (waveform A – the corresponding plots for the other experimental waveforms are shown in Additional file 1: Figures S17-S19). The results suggest that – consistent with the results obtained for synthetic nystagmus targets – the Pareto front estimate has converged by 100 generations for a population size of 4000, with larger population sizes (8000 or more) yielding comparable results.

**Fig. 6:**
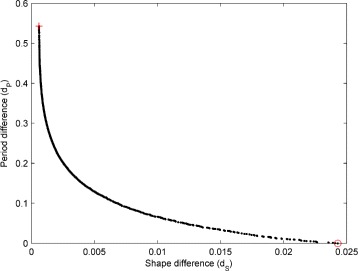
Example estimated Pareto front obtained by optimising the model to experimental nystagmus waveform A of Fig. 4. The *red circle* indicates the solution with the minimum period difference (equal to zero in this case) from the experimental oscillation, selected as the best individual; the corresponding fit to the experimental waveform can be seen in Fig. 11a. The *red cross* marks the solution with the minimum shape difference; the corresponding fit is shown in the left plot of Additional file 1: Figure S20A

**Fig. 7:**
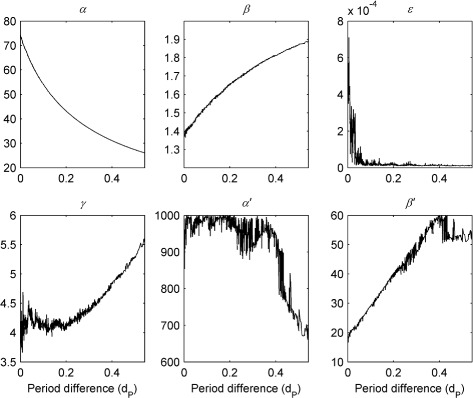
Variation in model parameters across the Pareto front shown in Fig. 6. As period difference *d*_*P*_ is increased, the front is traversed from *right* to *left* (i.e. from the *bottom right* corner of the shape-period plane indicated by the *red circle* to the *top left corner* indicated by the *red cross*)

Figure 6 shows a representative Pareto front estimate returned by NSGA-II for waveform A (the estimated fronts for waveforms B-D are very similar). The trade-off between the shape and period objectives is very clear from the plot: improved fits to the waveform period can only be obtained though poorer fits to the waveform shape and *vice versa* (cf. Figure 11 and Additional file 1: Figure S20). The evolution of the model parameters as the front is traversed can be see in Fig. 7. Interestingly, each parameter varies monotonically as the difference in period between the simulated and experimental waveform increases (or equivalently as the difference in shape decreases): on-response magnitude (*α* ^′^), off-response magnitude (*α*) and neural response time (*ε*) all decrease whilst on-response range (*β* ^′^), off-response range (*β*) and mutual inhibition strength (*γ*) all increase. The trade-off between period and shape fitting is thus directly reflected by a trade-off between the parameters controlling the shape of the saccadic neural response function: larger on- and off-response amplitudes combined with smaller on- and off-response ranges yield better fits to waveform period whilst smaller on- and off-response amplitudes combined with larger on- and off-response ranges yield better fits to waveform shape.

**Fig. 8:**
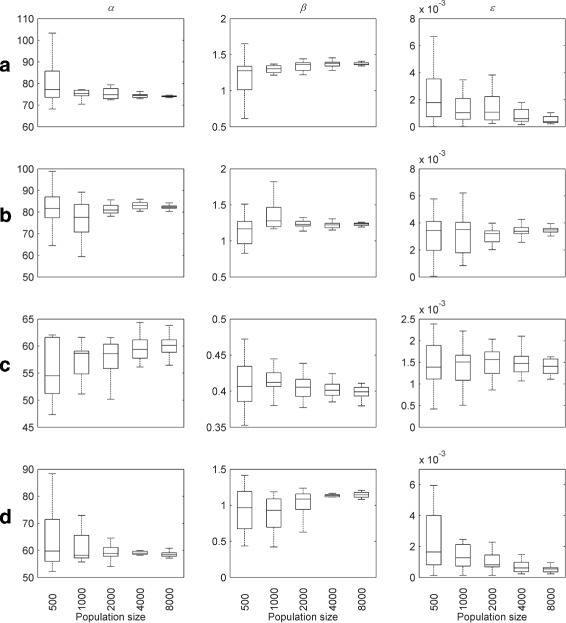
Optimised parameter values for experimental nystagmus waveforms as a function of population size. The first line of plots shows the optimised values of *α*,*β* and *ε* for waveform **a**, whereas those for **b**, **c** and **d** are shown by the second, third and fourth lines of plots, respectively. The *horizontal line* in each boxplot denotes the median parameter value. The edges of each box are the 25th and 75th percentiles. The whiskers extend to the interquartile range

**Fig. 9:**
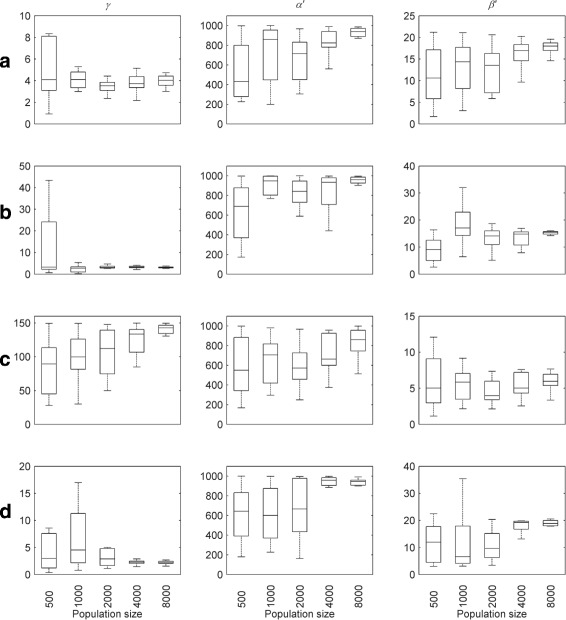
Optimised parameter values for experimental nystagmus waveforms as a function of population size. The first line of plots shows the optimised values of *γ*,*α* ^′^ and *β* ^′^ for waveform **a**, whereas those for **b**, **c** and **d** are shown by the second, third and fourth lines of plots, respectively. The *horizontal line* in each boxplot denotes the median parameter value. The edges of each box are the 25th and 75th percentiles. The whiskers extend to the interquartile range

**Fig. 10:**
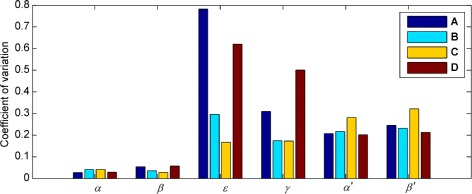
Optimising the model to experimental nystagmus waveforms: coefficients of variation of the parameters. Coefficients of variation were calculated from 16 NSGA-II runs with a population size of 4000 in each case (cf. Table 1)

For all waveforms, the corresponding optimised parameter values are shown in Figs. 8 and 9 for different population sizes. These confirm that good convergence is obtained for population sizes of 4000 and greater. Table 1 lists the mean optimised parameter values for this population size; the coefficients of variation are plotted in Fig. 10. Optimised parameter values for individual NSGA-II runs are listed in Additional file 1: Tables S4-S7.

**Table 1:** Optimised parameter values for experimental nystagmus waveforms

Waveform	*α*	*β*	*ε*	*γ*	*α* ^′^	*β* ^′^
A	74.0154 (0.02749)	1.3737 (0.05456)	0.0005 (0.7818)	3.9844 (0.30960)	913.0124 (0.2073)	17.6571 (0.2453)
B	82.2252 (0.04142)	1.2288 (0.03597)	0.0034 (0.2964)	3.1117 (0.17453)	929.9803 (0.2173)	14.8863 (0.2319)
C	60.2509 (0.04193)	0.3984 (0.02822)	0.00139 (0.1673)	139.0255 (0.17309)	815.4321 (0.2814)	5.8468 (0.3215)
D	58.6926 (0.02891)	1.1389 (0.05745)	0.0005 (0.6204)	2.3143 (0.50081)	919.9590 (0.2019)	18.4011 (0.2129)

Mean parameter values and coefficients of variation (shown in brackets) were calculated from 16 NSGA-II runs with a population size of 4000

Figure 10 shows that for waveforms A and D, the *ε* parameter has a much higher coefficient of variation than for waveforms B and C, suggesting that this particular waveform type (asymmetric pseudo-cycloid) is relatively insensitive to the value of *ε*. The coefficients of variation for the other parameters mirror those observed for synthetic nystagmus waveforms (cf. Figure 10 and Additional file 1: Figure S15). In particular, our results indicate that the fitness function is most sensitive to the parameters *α* and *β* that control the saccadic off-response (the saccadic braking signal).

**Fig. 11:**
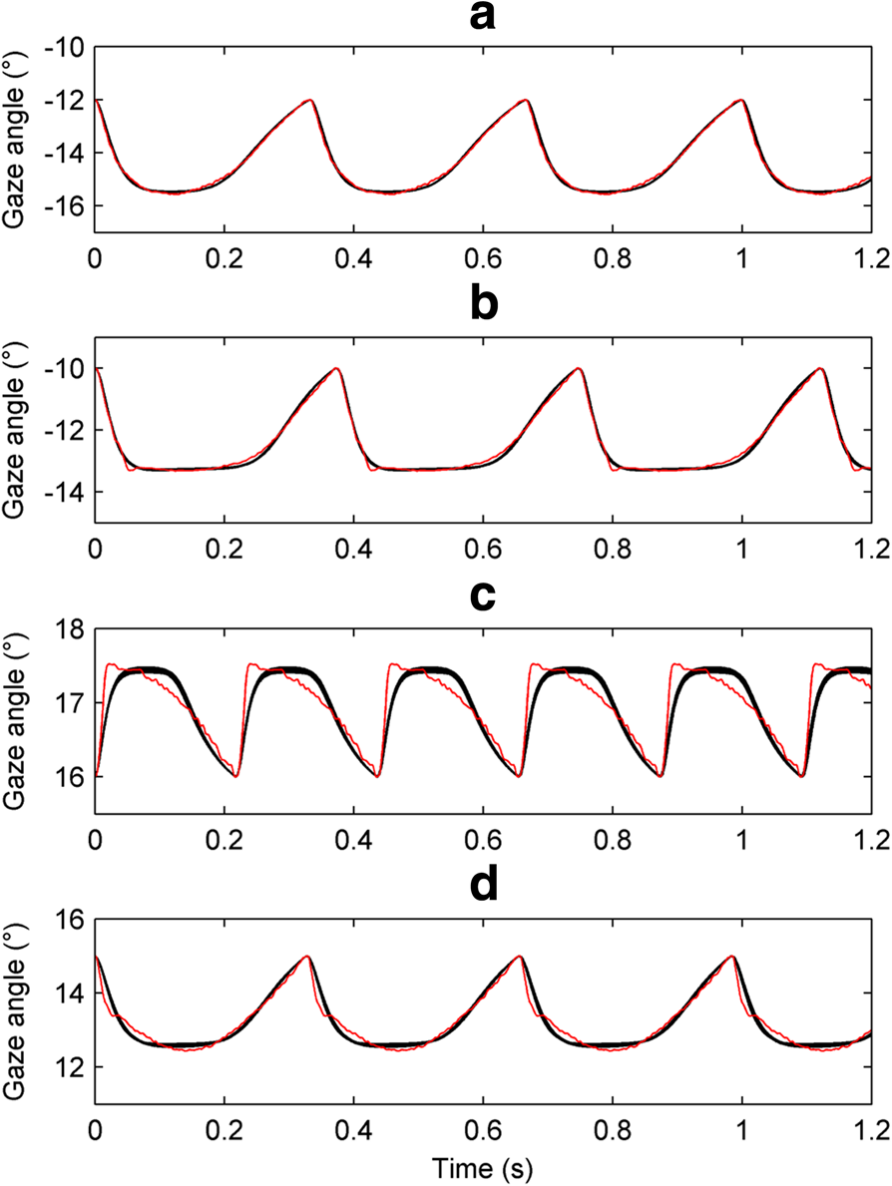
Fits of the model to experimental nystagmus waveforms. The target waveforms are plotted in *red*; the optimised waveforms obtained from 16 independent runs of NSGA-II with a population size of 4000 are plotted in *black*. In each plot, the vertical axis represents the horizontal gaze angle in degrees (°), with positive values denoting rightward eye positions. Time is in seconds (s). The nystagmus waveform types are: (**a**) asymmetric pseudo-cycloid; (**b**) jerk with extended foveation; (**c**) jerk; (**d**) asymmetric pseudo-cycloid

Finally, Fig. 11 compares the optimised and target waveforms. It can be seen that for target waveforms A, B and D, the optimised waveforms give a very good fit. In the case of waveform C, a poorer fit was obtained, as the model was unable to reproduce the very rapid fast phase. For this waveform, we increased the upper constraint of parameter *γ* to 150 because the optimised values obtained using an initial value of 12 hit this bound, suggesting that the optimal value might lay outside the initial range used. With this larger upper bound, the optimised *γ* parameter had a mean value of 139. However, this did not improve the fit. In addition, we observed that for all four waveforms fitted, the optimised values for parameter *α* ^′^ were concentrated fairly close to the upper constraint value of 1000 (see Fig. 9 and Table 1). This upper bound had been set directly on the basis of experimental measurements from primates, which implied an *α* ^′^ value around 800 [61]. We therefore performed exploratory runs with higher upper bounds for *α* ^′^, but did not obtain better fits to the waveform (results not shown).

### Fits to synthetic saccadic velocity profiles

Additional file 1: Figures S21-S24 show the convergence metrics (hypervolume indicator and minimum Pareto front distance) for fits of the model to synthetic saccadic velocity profiles. The boxplots in Additional file 1: Figures S25 and S26 plot the optimised parameter values for different population sizes (the plots for a population size of 500 are not shown as they provided inaccurate results). Taken together, these results indicate that for a population size of 8000, NSGA-II provides such good convergence that all the optimised parameter values are almost identical to the target values in each case (see Additional file 1: Table S2). The simulated velocity profiles generated using the optimised parameters are not shown as they were indistinguishable from the target ones.

As with nystagmus fitting, the accurate determination of the parameter values used to generate the synthetic data validated both the fitness function and the choice of optimisation algorithm.

### Fits to experimental saccadic velocity profiles

**Fig. 12:**
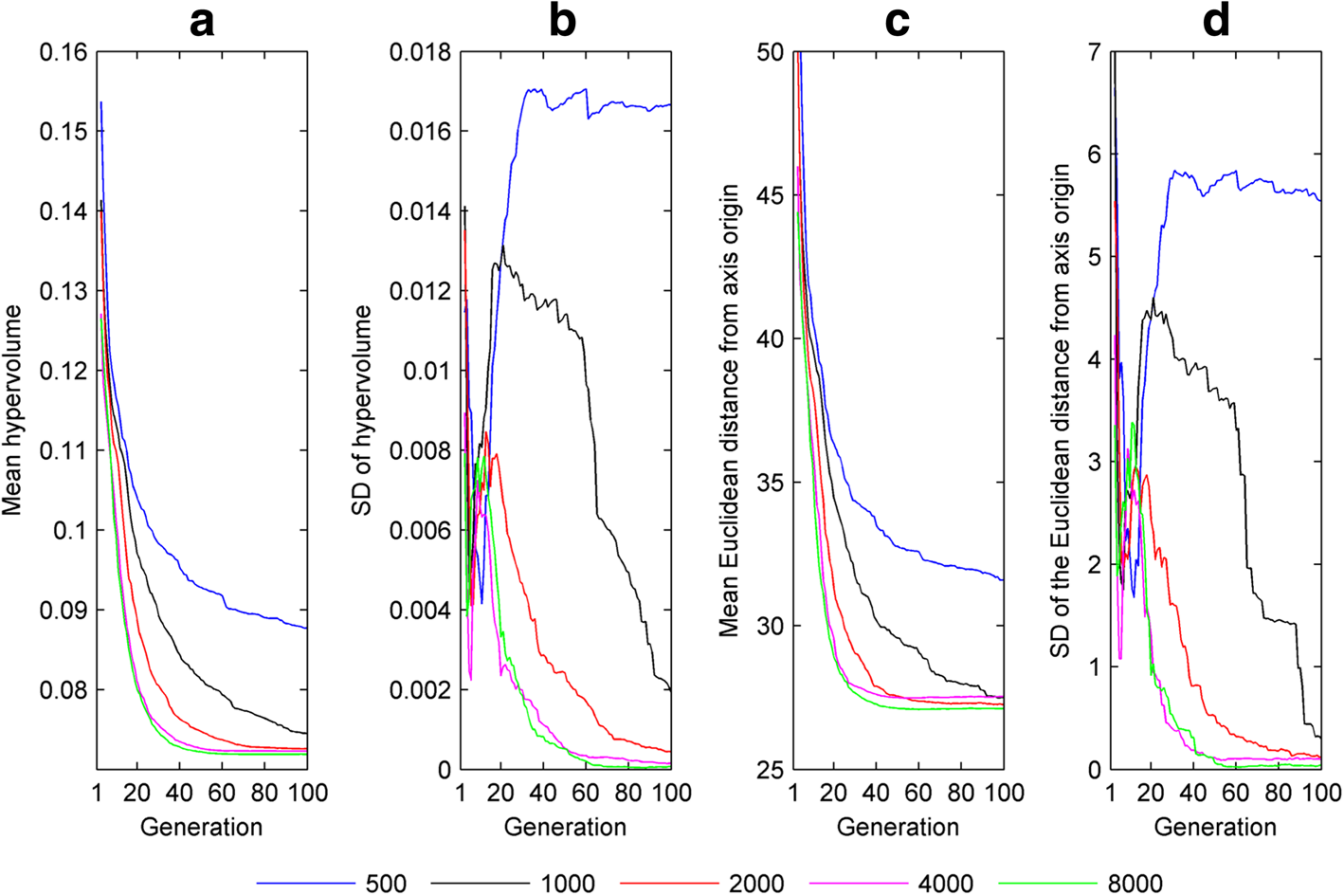
Convergence metrics of NSGA-II for fits to the experimental saccadic velocity profiles of Fig. 2. **a** Mean value of the hypervolume indicator $\mathcal {H}_{I}$ as a function of generation number *n*. **b** Standard deviation (SD) of $\mathcal {H}_{I}$ as a function of *n*. **c** Mean value of the smallest Euclidean distance $d_{\hat {\mathcal {F}}}$ between the Pareto front estimate and objective space origin as a function of *n*. **d** SD of $d_{\hat {\mathcal {F}}}$ as a function of *n*. Convergence metrics were calculated from 16 runs of NSGA-II each for the following population sizes: 500, 1000, 2000, 4000 and 8000

**Fig. 13:**
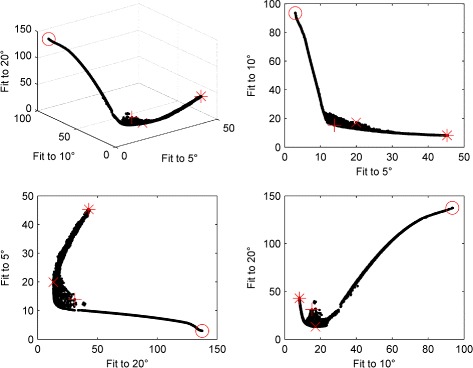
Estimated Pareto front obtained by optimising the model to the experimental saccadic velocity profiles of Fig. 2 with a population size of 8000. In each plot, the *red cross*, *red circle*, *red asterisk* and *red x* indicate the solutions yielding the minimum Euclidean distance to the axes origin, the best fit to a 5 degree saccade, the best fit to a 10 degree saccade and the best fit to a 20 degree saccade, respectively. Close-ups of each of these plots around the origin are shown in Additional file 1: Figure S27

For experimentally recorded saccadic velocity profiles, the convergence metrics show that – consistent with the synthetic profile fits – a population size of 8000 gave good convergence of the optimiser (see Fig. 12). Figure 13 shows an estimated Pareto front returned by NSGA-II. Projections of the three-dimensional front onto the different pairwise objective combinations are also plotted. As was observed for nystagmus fitting, there is a clear trade-off between objectives: improved fits to the velocity profile for a given saccade amplitude can only be achieved by degrading the quality of fit for one of the other amplitudes. However, it should be noted that the trade-off between fits to saccades of amplitude 10 and 20 degrees – the (10,20) trade-off – is not as pronounced as the (5,10) and (5,20) trade-offs, except in the neighbourhood of the origin (see Additional file 1: Figure S27). Indeed, away from this neighbourhood, the 10 degree and 20 degree objective functions appear to be cooperative.

**Fig. 14:**
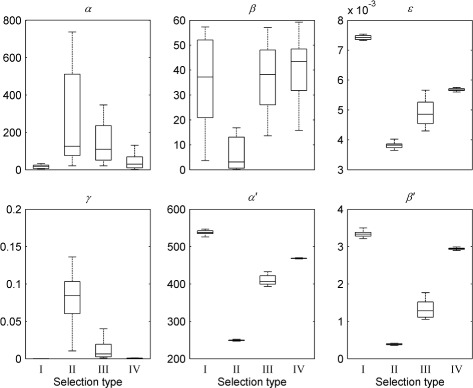
Optimised parameter values for experimental saccadic velocity profiles versus the method used for selecting the final solution. Method I selects the solution that minimises the Euclidean distance between the Pareto front population and the objective space origin; method II selects the best fit to a 5 deg saccade (objective 1); method III selects the best fit to a 10 deg saccade (objective 2); method IV selects the best fit to a 20 deg saccade (objective 3). The *top plots* (from *left* to *right*) show parameters *α*,*β* and *ε*, respectively; the *bottom plots* (from *left* to *right*) show parameters *γ*,*α* ^′^ and *β* ^′^, respectively. The *horizontal line* in each boxplot denotes the median parameter value. The edges of each box are the 25th and 75th percentiles. The whiskers extend to the interquartile range. All values shown were calculated from 16 NSGA runs with a population size of 8000

Interestingly, we found that the method used for choosing the final solution from the estimated Pareto front had a strong influence on the optimised parameter values obtained, with parameters *ε*, *α* ^′^ and *β* ^′^ exhibiting the greatest variation across selection methods (see Fig. 14 and Table 2).

**Table 2:** Optimised parameter values for experimental saccadic velocity profiles

Selection method	*α*	*β*	*ε*	*γ*	*α* ^′^	*β* ^′^
I	16.4500 (0.6088)	36.1980 (0.4769)	0.0074 (0.0094)	0.000034 (0.4659)	537.6500 (0.0112)	3.3400 (0.0232)
II	264.4100 (0.8873)	7.6514 (1.1634)	0.0038 (0.0255)	0.087352 (0.5497)	249.2100 (0.0052)	0.3900 (0.0942)
III	163.3700 (0.9756)	37.0826 (0.3589)	0.0049 (0.0846)	0.012519 (1.0388)	409.5300 (0.0316)	1.3200 (0.1752)
IV	42.3500 (0.8777)	39.2892 (0.3936)	0.0057 (0.0077)	0.000744 (0.2041)	468.5600 (0.0020)	2.9400 (0.0079)

Results are shown for each method used to select the final solution. Method I selects the solution that minimises the Euclidean

distance between the Pareto front population and the objective space origin; method II selects the best fit to a 5 deg saccade

(objective 1); method III selects the best fit to a 10 deg saccade (objective 2); method IV selects the best fit to a 20 deg saccade

(objective 3). Mean parameter values and coefficients of variation (shown in brackets) were calculated from 16 NSGA-II

runs with a population size of 8000. Optimised parameter values for individual NSGA-II runs are listed in Additional file 1: Tables S8-S11

**Fig. 15:**
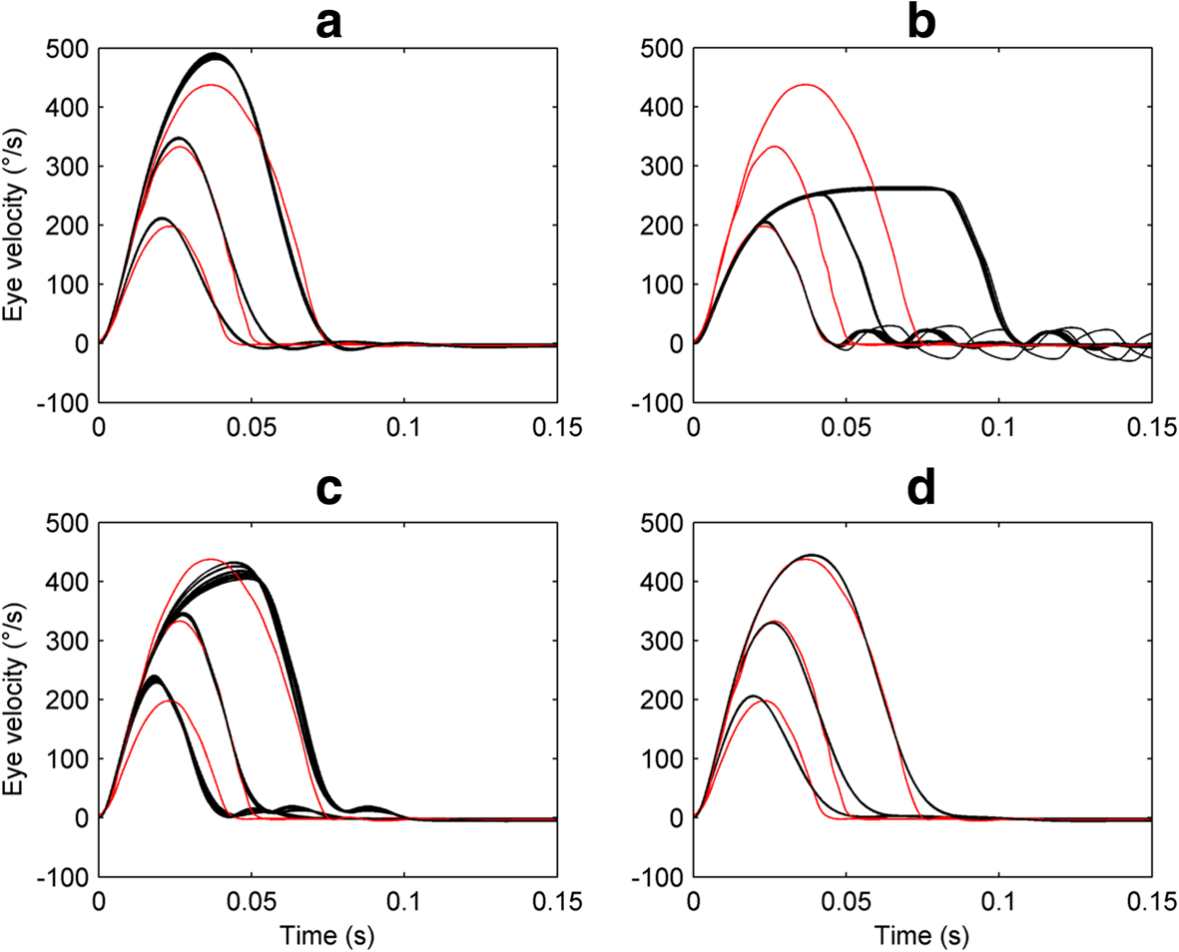
Fits of the oculomotor model to experimental saccadic velocity profiles. Each plot shows the optimised profiles obtained for saccade amplitudes of 5, 10 and 20 degs. using different methods for selecting the final solution. The target saccadic velocity profiles are plotted in *red*; the optimised saccadic velocity profiles obtained from 16 independent NSGA-II runs with a population size of 8000 are plotted in *black*. On each plot, the vertical axis represents the horizontal eye velocity in degrees per second (°/s), with positive values denoting rightward eye velocities. Time is in seconds (s). **a** Optimal fits obtained using selection method I (selects the solution that minimises the Euclidean distance of the Pareto front from the origin of objective space). **b** Optimal fits obtained using method II (best fit to a 5 deg. saccade). **c** Optimal fits obtained using method III (best fit to a 10 deg. saccade). **d** Optimal fits obtained using method IV (best fit to a 20 deg. saccade)

Figure 15 compares the optimised saccadic velocity profiles obtained using the different selection methods to the target experimental data, while Table 3 shows the corresponding fitness values for each objective. It can be seen that no single selection method (and hence no single parameter set) gave good fits to the velocity profiles for all saccade amplitudes simultaneously. In particular, while – as expected – methods II, III and IV produced good fits for their corresponding amplitudes, method I gave a poor fit to the largest amplitude saccade.

**Table 3:** Optimising the model to experimental saccadic velocity profiles: fitness values on individual objectives

Selection method	5 ^∘^	10 ^∘^	20 ^∘^
I	16.6687 (0.0229)	16.5143 (0.0190)	13.9354 (0.0125)
II	3.1672 (0.0285)	94.7590 (0.0072)	138.2400 (0.0044)
III	40.7405 (0.1000)	8.7522 (0.0557)	36.6450 (0.1692)
IV	19.1386 (0.0202)	17.3887 (0.0106)	13.1121 (0.0013)

Results are shown for each method used to select the final solution. Method I selects the solution that minimises the Euclidean

distance between the Pareto front and the objective space origin; method II selects the best fit to a 5 deg saccade (objective 1);

method III selects the best fit to a 10 deg saccade (objective 2); and method IV selects the best fit to a 20 deg saccade

(objective 3). Mean fitness values and coefficients of variation (shown in brackets) were calculated from 16 runs of NSGA-II

for a population size of 8000. Fitness values were normalised by the number of points in each velocity profile

### Speedup obtained using the multiple NSGA-II parallel method

To evaluate the speedup obtained on each of the computer hardware systems tested, we calculated the execution time of the parallel ODE solver (described in Additional file 1) as a function of the number of model integrations for each system in turn. Here, we define a single model integration (or orbit) to be the numerical integration of the oculomotor model over a fixed, simulated time interval (6 s) for one parameter combination.

**Fig. 16:**
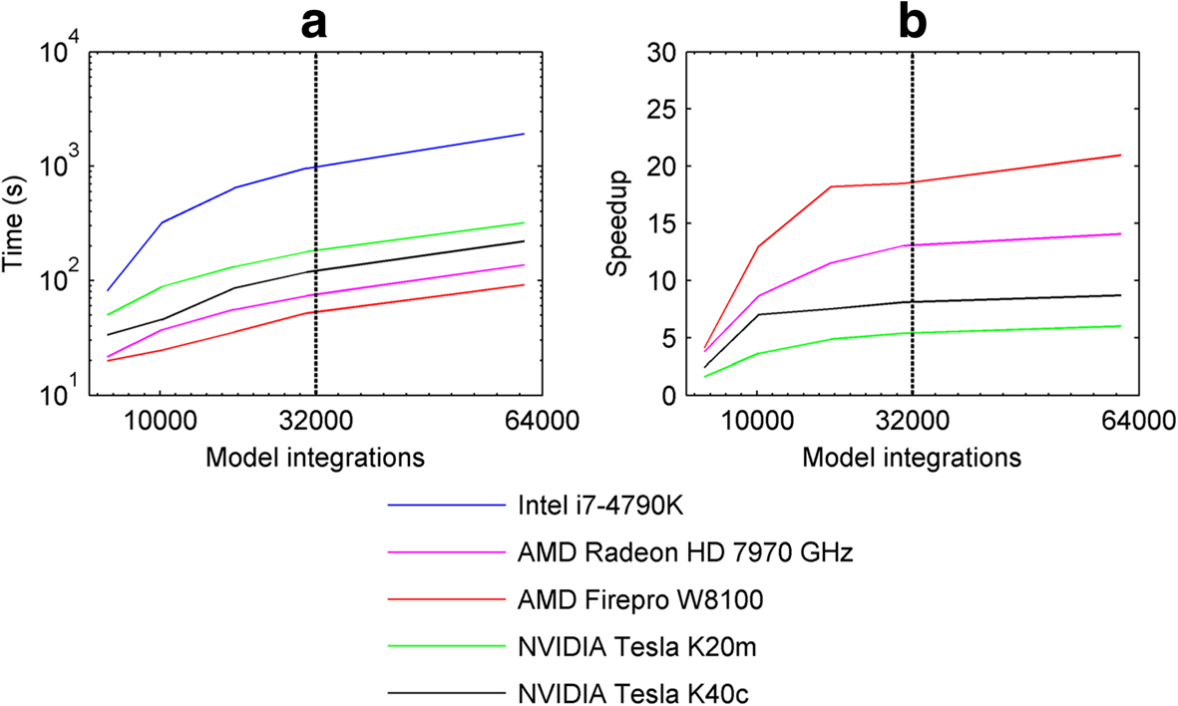
CPU/GPU execution times and speedup. **a** CPU and GPU execution time in seconds (s) vs the number of model integrations. **b** CPU:GPU execution time ratio (speedup) vs the number of model integrations. The numerical solver used was the implicit mid-point method (time step *Δ* *t*=5×10^−6^ s) and the simulated model time was 6 s. The model parameters were uniformly distributed in the following ranges: *α*∈ [ 1,1000];*β*∈ [ 0.001,10];*ε*∈ [ 0.00001,0.1];*γ*∈ [ 0.00001,12];*α* ^′^∈ [ 1,1000];*β* ^′^∈ [ 0.001,60]. For each integration, the initial motor error *m* was set to 2 degrees; all other variables were initially set to 0. In each plot, the *dotted line* denotes 32000 integrations

Figure 16 a compares the execution time of the parallel ODE solver program when using the CPU compared to when using each of the four GPU cards. The corresponding CPU:GPU execution time ratio in each case is shown in Fig. 16 b. It can be seen that all the GPU cards provided a significant speedup compared to the Intel Core i7-4790K CPU. The best performance was obtained using the AMD FirePRO W8100, which gave a speedup of around 20 at 32000 parallel model integrations.

We note that these results should not be considered as conclusive performance comparisons for these particular models, because our implementation of the parallel ODE solver is not yet fully optimised to run on GPUs. However, the results do suggest that the AMD GPUs we tested provide better performance compared to the NVIDIA GPUs for our particular task, and that converting serial C code to GPU parallel code (OpenCL) can provide considerable speedups on AMD hardware. Based on preliminary tests, one possible explanation for the differences in execution time we report here could be that the AMD cards are not affected by thread divergence as much as the NVIDIA cards. It should be noted, though, that even the GPU which performed worst in our test (the NVIDIA Tesla K20m) gave a speedup of more than 5.

**Fig. 17:**
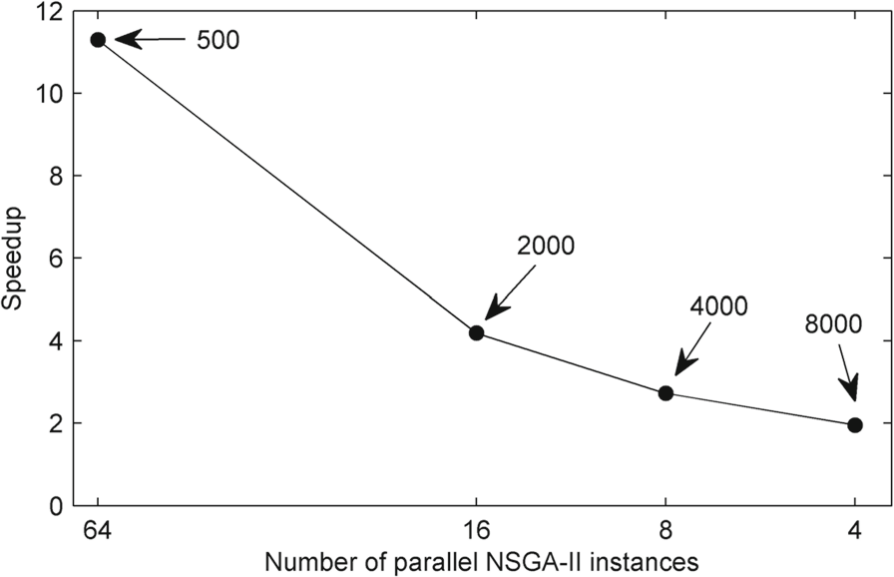
Speedup obtained with the parallel NSGA-II method versus the number of parallel instances using the AMD Firepro W8100 GPU. Speedup is defined as the ratio of parallel execution time to serial execution time for the same number of NSGA-II instances. The indexed numbers indicate the population size for each NSGA-II instance. In each case, the sum of the populations of all NSGA-IIs running in parallel is 32000; this is in the range of parallel integrations that provides the maximum speedup, compared to the CPU (see Fig. 16)

For all the GPUs we tested, the maximum speedup observed saturated when the number of model orbits exceeded 32000 (see Fig. 16 b). Hence, to assess how to best exploit this speedup using our multiple NSGA-II parallel method, we examined the effect of reducing the number of NSGA-II instances *n*_*I*_, whilst increasing the population size *N* of each instance so as to keep the total number of simulations *n*_*P*_=*n*_*I*_ *N* fixed at 32000. The different *n*_*I*_ values used were 64, 16, 8 and 4, corresponding to the population sizes 500, 2000, 4000 and 8000, respectively. For each choice of *n*_*I*_, we compared the execution times obtained by running the NSGA-II instances in parallel on the GPU to those obtained by running them serially. Figure 17 shows that whilst a significant parallel:serial speedup is obtained for all *n*_*I*_ values, the speedup decreases as the number of parallel instances decreases (or equivalently as the population size increases). This was as anticipated, because larger population sizes use more of the GPU capabilities when a single NSGA-II instance is run. It should also be noted that the population size and number of parallel instances that can be used are constrained by the specifications of the GPU card and the system’s RAM. Our test system had 32 GB of RAM, of which 6 GB was apportioned to the RAM disk.

The significant acceleration in the optimisation process that resulted from parallelising the model integrations on a GPU can be seen in Table 4, which compares parallel and serial NSGA-II execution times for fits of the model to experimental nystagmus oscillations. In particular, it should be noted that fitting all 4 waveforms using 16 NSGA-II runs with a population of 4000 took approximately 34 hours using a combination of the FirePRO W8100 GPU and i7-4790K CPU, compared with 577 hours using the i7-4790K CPU alone, a speedup of over 16.

**Table 4:** Comparison of serial and parallel NGSA-II execution times

Population	Serial-CPU (1 core)	Parallel-CPU (4 cores)	Parallel-GPU
500	281.2	70.4	3.9
1000	450.8	113.1	6.5
2000	1077.7	270.3	15.8
4000	2299.3	577.3	34.4
8000	6780.3	1709.2	110.2

Each column shows the time required in hours to optimise the model 16 times to all 4 experimental nystagmus waveforms,

as population size is increased. NSGA-II was run for 100 generations in each case. Serial-CPU: results obtained using only

1 core of the i7-4790K CPU. Parallel-CPU: results obtained using all 4 cores of the i7-4790K CPU. Parallel-GPU: results

obtained using the FirePRO W8100 GPU, with all 4 cores of the i7-4790K CPU

## Discussion

The aetiology of infantile nystagmus is not yet well understood and a number of computational models have been proposed to gain deeper insights into the disorder. The use of parameter optimisation methods greatly facilitates the development of such models by enabling them to be systematically tested and analysed, thereby providing specific directions on how a given model can be modified to fit a broader range of experimental data.

Here, we have presented the results of using a well-established multi-objective genetic algorithm, NSGA-II, to fit the oculomotor model of Broomhead et al. [22] to experimental infantile nystagmus and saccadic time series data (nystagmus oscillations and normal saccades, respectively). We carefully tuned the parameters of the genetic algorithm, particularly population size, by performing test runs on synthetic datasets chosen to qualitatively match characteristic properties of their experimental counterparts. The synthetic datasets also assisted us in the selection of appropriate fitness functions to measure the goodness-of-fit of the model to the target time series. The final fitness functions used were based on matching shape and period for nystagmus oscillations and velocity profiles for saccades. For fits to nystagmus data, unstable periodic orbit analysis proved to be a highly effective method for extracting single oscillation cycles, being robust with respect to both intra-subject and inter-subject waveform variability.

To make the optimisation process computationally tractable, we executed the GA on a hybrid CPU-GPU architecture. Our hybrid method used parallel runs of independent instances of the NSGA-II optimiser, where the most computationally expensive task – the integration of the oculomotor model – was performed by the compute units of a GPU, under the control of a CPU which also implemented the genetic operations and fitness evaluation. This parallel master-slave method enabled multiple NSGA-II instances to be run on a single GPU, leading to an order of magnitude speedup, depending on the NSGA-II population size and the particular GPU card used (Figs. 16-17 and Table 4). Our method provided us with comparable acceleration to an HPC cluster, yielding a maximum speedup of ≈20 (using the AMD Firepro W8100) compared to a high-end CPU (the Intel Core i7-4790K).

We note that although we obtained very good results with NSGA-II – being able to successfully fit the model to different experimental targets – there are other multi-objective methods that may provide comparable results. For example, the *ε*-constraint method could be used, although this would require the constraint value of each objective to be explored in order to generate the Pareto front for each experimental target [40]. By comparison, each single run of NSGA-II yields an approximation to the full Pareto front. A hybrid method combining NSGA-II and a local optimiser (e.g. simulated annealing) could also be applied. Furthermore, more sophisticated methods (such as CRS-tuning [83]) could be used to select the parameters of NSGA-II, besides brute force. A comparison of the accuracy of the fits obtained using different optimisers – and the number of function evaluations required in each case – would be an interesting follow-up study.

The results of applying our optimisation method to experimental data quantified some of the strengths and weaknesses of the oculomotor model in simulating normal and abnormal eye movements, whilst also providing possible insights into the underlying physiology. In the case of nystagmus fitting, the model could accurately replicate a number of oscillation types (e.g. jerk, asymmetric pseudo-cycloid and pseudo-cycloid – Fig. 11). However, some oscillations (e.g. jerk with a very rapid fast phase – Fig. 11c) and some characteristics of specific oscillations (such as the transition between the fast phase and slow phase in Fig. 11b) could not be exactly reproduced. Furthermore, the optimised parameter distributions indicated that the fits to data are most sensitive to the model parameters *α* and *β* (Fig. 10 and Additional file: Figure S15), which control the magnitude and operational range of the saccadic braking signal (the saccadic off-response), respectively. This finding is consistent with the hypothesis of van Gisbergen et al. [61], Broomhead et al. [22] and Akman et al. [18] that the dynamics of the braking signal is the cause of the oculomotor instability that precedes the onset of endogenous oscillations. In addition, the consistently high optimised values of the *α* ^′^ parameter (Fig. 9 and Table 1), which controls the magnitude of the saccadic on-response, may indicate that the burst neurons have a higher average maximal firing rate in human subjects than in primates.

In the case of saccadic data, although the velocity profiles for each saccade amplitude could be individually fitted with different parameter sets, no single parameter set was able to fit all the profiles simultaneously, despite our use of a multi-objective optimiser (Fig. 15 and Table 3). One possible explanation for this deficiency of the model may relate to the way in which the mutual inhibition between the right and left burst neuron populations is currently represented. Figure 14 and Table 2 show that the optimised value of the parameter *γ* controlling the strength of this mutual inhibition decreases rapidly to near-zero as the saccade amplitude is increased from 5 degrees. It follows that for the Broomhead model, 5 degree saccades may belong to a different fitting class than larger saccades, as is further implied by the relatively weak trade-off between fits to saccades of amplitude 10 and 20 degrees (Fig. 13). This could suggest, for example, modifying the model to incorporate gaze angle-dependent mutual inhibition.

The predictive capacity of the model could be further improved by modifying the neural integrator equation in line with the experimental findings of Khojasteh et al. [84], who have shown that in horizontal eye movements there is a large variability in the neural integrator time constant and the location of the null zone. Initially, future versions of the model could include the neural integrator time constant as a parameter. Subsequently, a better equation describing the experimental data in more detail could be developed that also simulated the variability in null zone position between individuals.

## Conclusions

In this study, we have presented a GPU-accelerated method for fitting the saccadic model of Broomhead et al. [22] to experimental infantile nystagmus and saccadic data sets. We anticipate that our optimisation method will enable us to examine how the parameters of oculomotor models evolve over the duration of a nystagmus time series recording. During a single experimental recording, the nystagmus waveform characteristics can change due, for example, to variations in attention level and gaze angle [5]. Optimising the model to successive segments of such recordings would trace out a path in the model’s parameter space, directly relating the observed transitions between different oscillation types to the model’s bifurcation structure. Furthermore, the association of nystagmat groups with specific regions of parameter space could potentially help identify the mechanisms underlying the onset of oscillations in each group.

More generally we believe that the methodology we used to develop the fitness functions and to tune the NSGA-II parameters could be used to optimise any similar biological model to data, thereby allowing its predictive capacity to be comprehensively explored. Indeed, we are currently using our techniques to accelerate the optimisation of spiking models of the cardiac action potential and biochemical models of oscillatory gene expression. In terms of attaining broader applicability of our methods, the parallel ODE solver suite could be further expanded to include additional numerical methods, capable of integrating different classes of ODE model (e.g. variable step and multi-step methods – this would also accelerate the integration of the saccadic model considered here and its future iterations). In addition, a better interface between the parallel ODE solvers and MATLAB could be obtained by using a.mex file. This would enable the GPU to be called directly without running an external executable file and would also allow users to run the solvers without requiring a RAM disk to save their results.

Finally, future applications of our optimisation protocol will require the researcher to compare the model execution time to that of the other optimisation processes and decide whether additional parallelisation is necessary. In our case, for example, the execution time of the NSGA-II operations increases considerably for population sizes greater than 10000 (see Additional file: Figure S7). This is as expected because NSGA-II is of *O*(*MN* ^2^) computational complexity, where *M* is the number of objectives and *N* is the population size [57]. The profiler output shows that the MATLAB function which causes this increase is nonDominatedRank.m, which ranks the individuals in the population. If there was a requirement for further reduction in NSGA-II execution time, an accelerated version of the ranking function that runs on a GPU could be developed.

## Additional file

Additional file 1 Contains Supplementary Figures S1-S27 and Supplementary Tables S1-S11. (PDF 5998 kb)
