# Supplementary material for: Optimisation of an exemplar oculomotor model using multi-objective genetic algorithms executed on a GPU-CPU combination
Source: BMC Syst Biol. 2017 Mar 24;11:40. doi: 10.1186/s12918-017-0416-2 (PMC5364688; doi:10.1186/s12918-017-0416-2)
Supplement: Additional file 1 — Contains Supplementary Figures S1-S27 and Supplementary Tables S1-S11. (PDF 5998 kb) [file 12918_2017_416_MOESM1_ESM.pdf]

Supplementary Information

Optimisation of an exemplar oculomotor model using multi-objective genetic algorithms executed on a GPU-CPU combination, Avramidis & Akman

Contents

The GPU executable **1**

Supplementary Figures **2**

Supplementary Tables **19**

References **22**

The GPU executable

Programs that are executed on a GPU have to be written using a specific programming framework. The two most widely used frameworks are the NVIDIA Compute Unified Device Architecture (CUDA) [1] and the Open Computing Language (OpenCL) [2]. The CUDA framework is a proprietary architecture specifically designed to be run only on NVIDIA GPUs. By contrast, OpenCL which is managed by the Khronos Group [3], is an open framework which can be used to program almost any parallel architecture, such as multicore CPUs, GPUs, coprocessors (e.g. the Intel Xeon Phi) or a network of connected GPUs and/or CPUs. To ensure better hardware compatibility of our code, we therefore developed our executable in the OpenCL framework.

OpenCL includes a C99 based language for writing functions (kernels), which are executed on OpenCL devices (e.g. CPUs or GPUs) and an application programming interface (API). The API is used to define and control the OpenCL devices and execute the kernels on them. A simple OpenCL program involves the steps of finding the computing device, compiling the code that will be run on the device, copying the data to the device, performing the computation and copying back the results of the computation.

Our OpenCL program includes a kernel with functions that numerically solve the saccadic model for different parameter combinations. Moreover, our program includes code written in C++, which, using the OpenCL API, performs the actions necessary to run the kernel on the GPU. The numerical method that we used in the kernel was the implicit mid-point rule [4], with a time step $\Delta t = 5 \times 10^{-6}$ (as the saccadic model is a stiff system, a small time step is required for accurate integration). To assess whether the ODE solver and the selected time step gave accurate results, we compared the model solutions generated using our method with those obtained using the MATLAB stiff ODE solver ode15s, with relative and absolute tolerances both set to 10^{-8}.

The logical flow of the GPU executable program is as follows. First, the program reads the input binary file which contains the parameter combinations comprising the NSGA-II population and the initial conditions. Subsequently, these parameter values and initial conditions are transferred to the GPU's RAM. Next, the OpenCL kernel is called to integrate the model for 80 time steps. In parallel with the integration (the current kernel call), the results of the previous integration (the previous kernel call) are written to a binary file. Once the model has been integrated over the required timespan for all parameter combinations, the program quits.

We found that the sampling frequency of the results (i.e. the number of points in the time series used for calculating fitness) played an important role in the GPU computation time when fitting the model to nystagmus waveforms. This was due to the bottleneck caused by the transfer to and from the GPU memory, and the time required to write the data to the binary file. Higher sampling frequencies substantially increased the time required for model integration and solution analysis (fitness evaluation) and also the size of the binary file produced. For example, using a sampling frequency of 2500 Hz, 20000 different parameter sets with a simulation time of 6 s each produced a 2.23 GB file, with an overall computation time of 35.44 s. Increasing the sampling frequency to 5000 Hz increased the file size to 4.46 GB and the overall computation time to 45.56 s.

However, it was not possible to overcome this problem by arbitrarily reducing the sampling rate, since by considering fits to synthetic nystagmus waveforms (see Figure S4), we found that a low sampling frequency did not allow accurate period and amplitude measures to be obtained. In order to choose the lowest sampling frequency capable of resolving this speed/accuracy trade-off, we generated very highly sampled versions of the synthetic waveforms in Figure S4 by integrating the model for each corresponding parameter set with Δt set to 5×10^{-6} s (i.e. a sampling frequency of 5×10^{6} Hz). We then subsampled each time series at 250, 500, 1000, 1250, 2500 and 5000 Hz to obtain a set of candidate waveforms. Next, we calculated the sum-of-square errors for amplitude and period between the highly sampled and candidate waveforms. On the basis of these results, the final sampling frequency was chosen to be 2500 Hz, as using a higher frequency caused no significant reduction in error. Returning results at this frequency enabled us to minimise the effect of the GPU call bottleneck, the amount of memory used and the computation time required to analyse the results, without significantly degrading the accuracy of the period and amplitude calculations.

Supplementary Figures

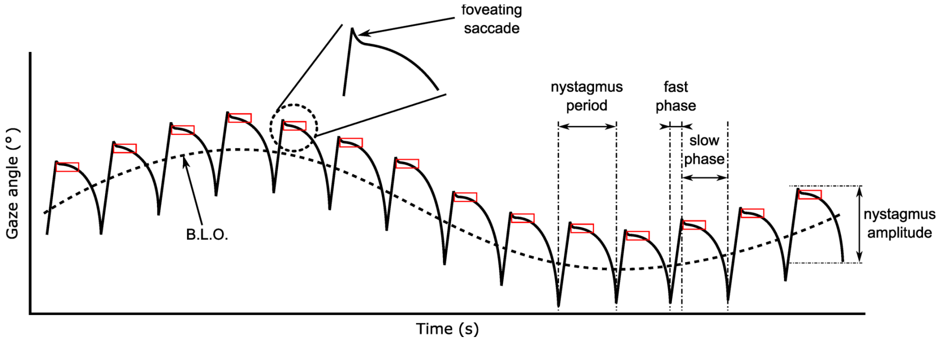

Figure S1: **Schematic illustration of a unidirectional jerk infantile nystagmus waveform with its key characteristics highlighted.** B.L.O.: baseline oscillation. The red rectangles indicate foveation windows. Time is given in seconds (s). Horizontal gaze angle is in degrees (°). Figure adapted from [5].

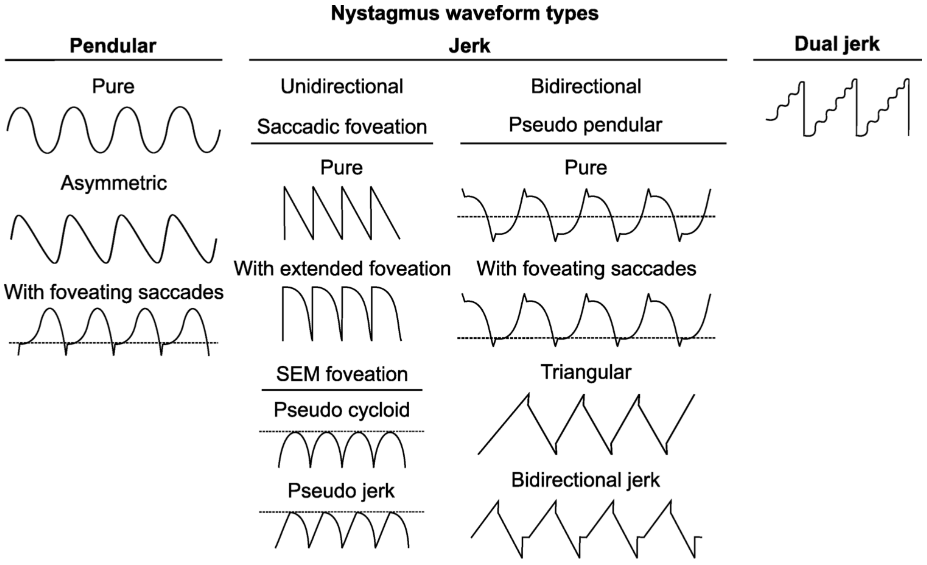

Figure S2: **Qualitative characteristics of different infantile nystagmus waveform types.** The dashed line represents the fixation point. SEM: slow eye movement. Figure adapted from [6].

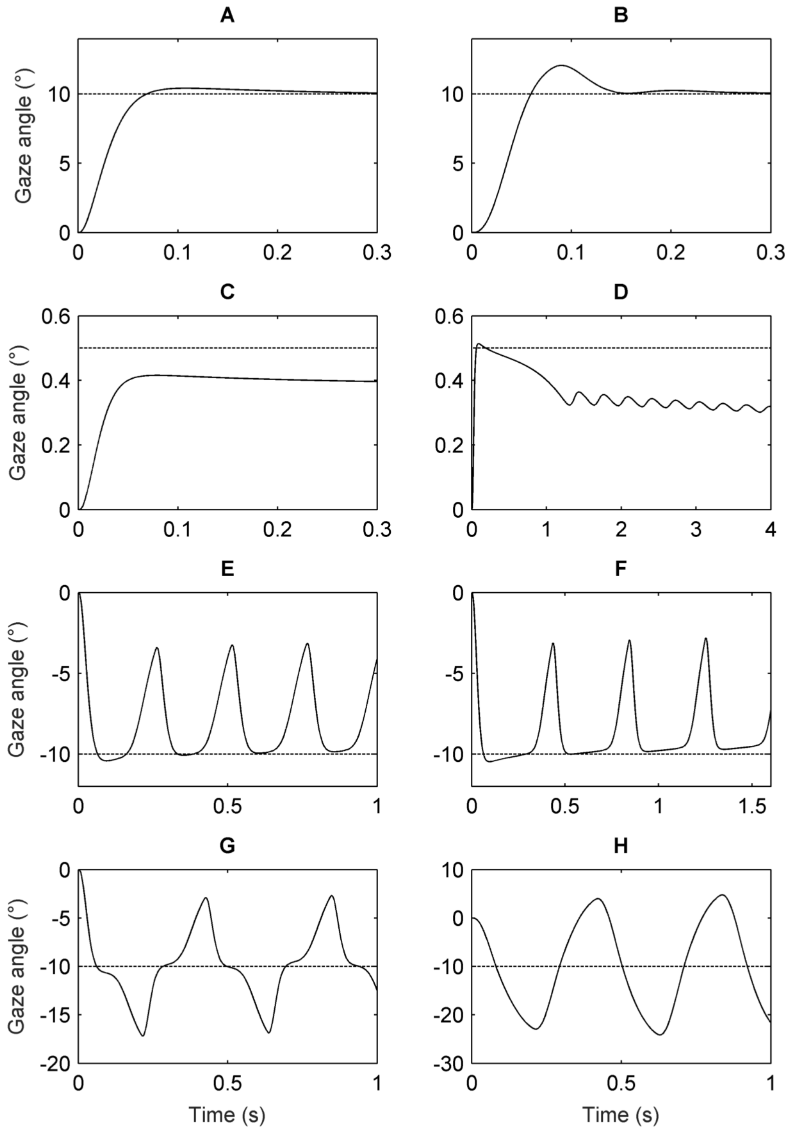

Figure S3: **Range of eye movements simulated by the oculomotor model of Broomhead** *et al.* Time series were generated using different parameter choices for α, β and ϵ, with initial conditions set to $g(0) = n(0) = r(0) = l(0) = 0$ and $m(0) = \Delta g$, simulating a saccade of Δg degrees executed from rest at the primary position (0 degrees). The values of γ, α' and β' were fixed at 0.05, 600 and 9, respectively. (A) Normometric saccade: α=20, β=3, ϵ=0.001, Δg=10. (B) Dynamic overshoot: $\alpha = 20$, $\beta = 3$, $\epsilon = 0.015$, Δg= 10. (C) Hypometric saccade: $\alpha = 206$, $\beta = 3$, $\epsilon = 0.001$, $\Delta g = 0.5$. (D) Small-amplitude nystagmus: $\alpha = 207.656$, $\beta = 3$, $\epsilon = 0.006$, $\Delta g = 0.5$. (E) Jerk IN: $\alpha = 240$, $\beta = 3$, $\epsilon = 0.004$, Δg = -10. (F) Jerk with extended foveation: $\alpha = 240$, $\beta = 3$, $\epsilon = 0.0048$, Δg = -10. (G) Bidirectional jerk IN: $\alpha = 240$, $\beta = 3$, $\epsilon = 0.006$, Δg = -10. (H) Pendular IN: α = 240, $\beta = 3$, $\epsilon = 0.06$, Δg = -10. In each plot, the vertical axis represents horizontal gaze angle in degrees (°), with positive (negative) values representing rightward (leftward) gaze, respectively. Time is in seconds (s). The dashed lines represent the target position.

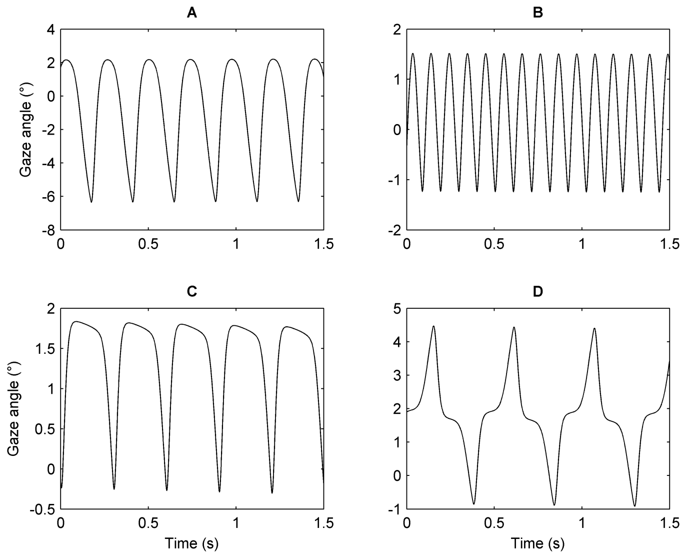

Figure S4: **Synthetic infantile nystagmus waveforms.** The model parameter values used to generate each oscillation are shown in Table S1. The initial conditions were set to $g(0) = n(0) = r(0) = l(0) = 0$ and $m(0) = 2$ in each case. The simulated waveform types are: asymmetric pseudo-cycloid (A); pseudo-cycloid (B); jerk (C); and bidirectional jerk (D). On each plot, the vertical axis represents the horizontal gaze angle in degrees ($^\circ$), with positive values denoting rightward eye positions. Time is in seconds (s).

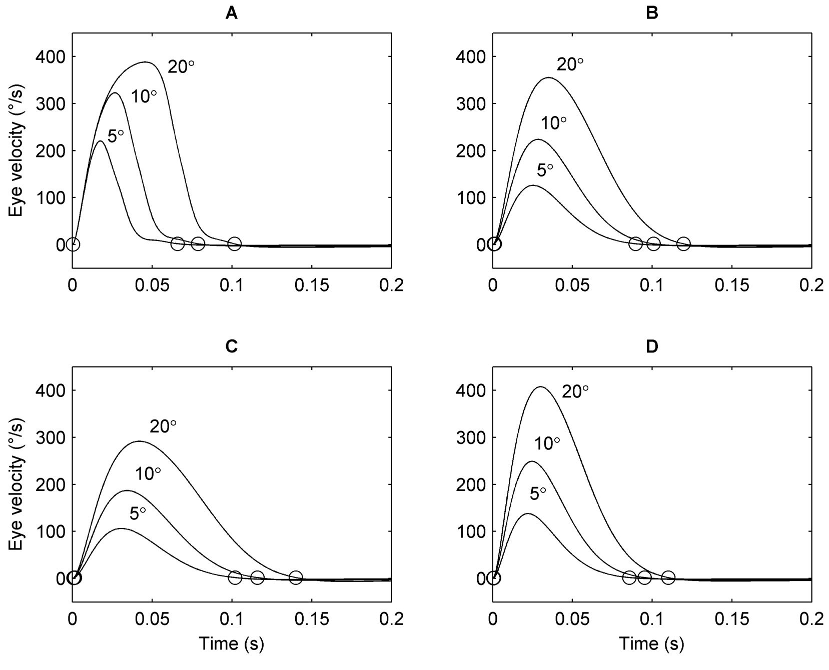

Figure S5: **Synthetic saccadic velocity profiles.** Velocity profiles were simulated using the model parameter values listed in Table S2 for amplitudes of 5, 10 and 20 degrees. For each amplitude Δg, the initial conditions were set to $g(0) = n(0) = r(0) = l(0) = 0$ and $m(0) = \Delta g$. The black circles indicate the initiation and termination of each saccade, calculated by applying a velocity threshold of 2 deg/s. On each plot, the vertical axis represents the horizontal eye velocity in deg/s ($^\circ$/s), with positive values denoting rightward motion. Time is in seconds (s).

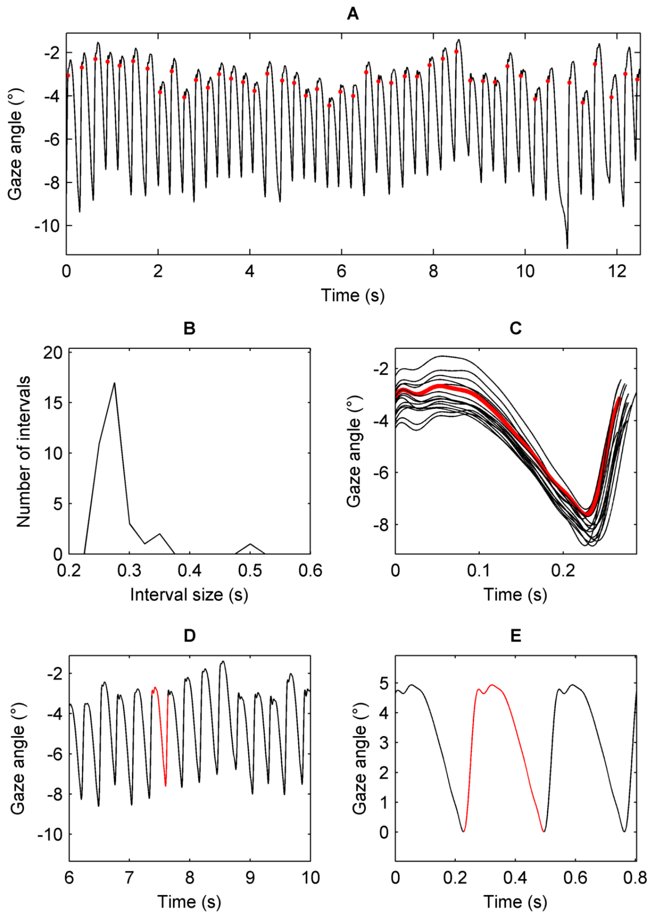

Figure S6: **Key stages of the unstable periodic orbit (UPO) extraction method.** (A) Experimental time series of a right-beating jerk nystagmus. Red points (velocity threshold of 70 deg/s) delineate the different inter-cycle intervals τ_k. (B) Histogram of the transformed interval data, showing a clear peak at $\tau_* = 0.28$s. (C) Candidate periodic orbits extracted from the time series by thresholding $|\tau_k - \tau_*|$. The UPO selected for further processing is shown in red. (D) Part of the experimental recording shown in A. The extracted UPO is shown in red. (E) 3 concatenated copies of the extracted UPO. The final UPO used for parameter optimisation, which starts at the beginning of the fast phase, is shown in red. Gaze angle is in degrees (°); time is in seconds (s).

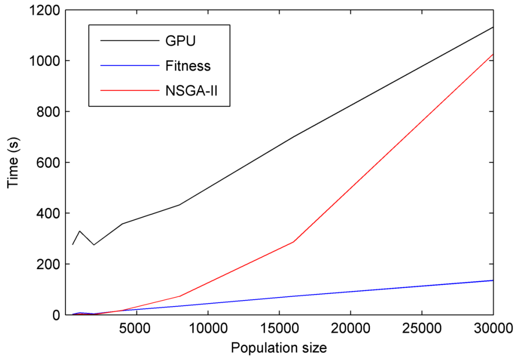

Figure S7: **Mean execution time of three key computational tasks in an optimisation run as a function of population size.** Execution time was computed for the following tasks: (i) model integration (GPU executable); (ii) fitness function evaluation; and (iii) NSGA-II operations. Mean values were taken from 8 optimisation runs for 5 generations each. The variation in execution times for each run with the same population size is negligible and is therefore not shown.

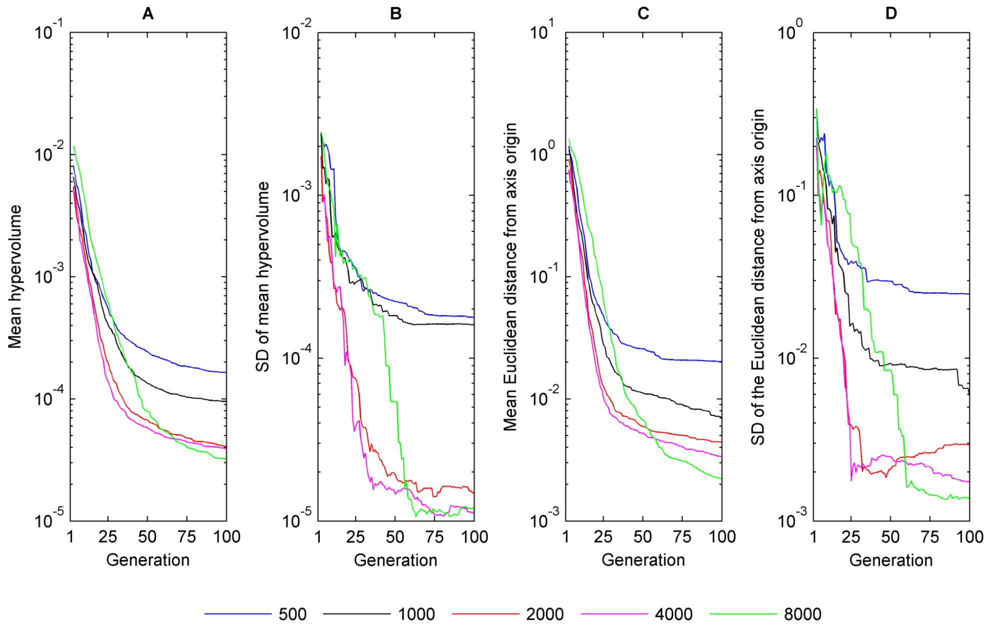

Figure S8: **Convergence metrics of NSGA-II when fitting the model to synthetic nystagmus waveform A of Fig. S4.** (A) Mean value of the hypervolume indicator $\mathcal{H}_I$ as a function of generation number n. (B) Standard deviation (SD) of $\mathcal{H}_I$ as a function of n. (C) Mean value of the smallest Euclidean distance $d_{\hat{\mathcal{F}}}$ between the Pareto front estimate and objective space origin as a function of n. (D) SD of $d_{\hat{\mathcal{F}}}$ as a function of n. Convergence metrics were calculated from 16 runs of NSGA-II each for the following population sizes: 500, 1000, 2000, 4000 and 8000.

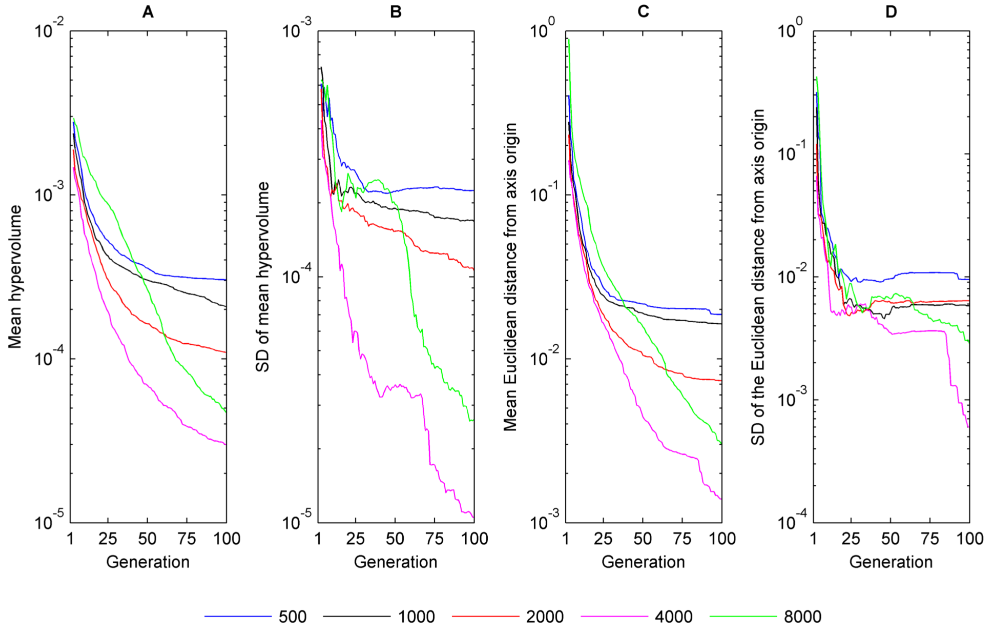

Figure S9: **Convergence metrics of NSGA-II when fitting the model to synthetic nystagmus waveform B of Fig. S4.** (A) Mean value of the hypervolume indicator $\mathcal{H}_I$ as a function of generation number n. (B) Standard deviation (SD) of $\mathcal{H}_I$ as a function of n. (C) Mean value of the smallest Euclidean distance $d_{\hat{\mathcal{F}}}$ between the Pareto front estimate and objective space origin as a function of n. (D) SD of $d_{\hat{\mathcal{F}}}$ as a function of n. Convergence metrics were calculated from 16 runs of NSGA-II each for the following population sizes: 500, 1000, 2000, 4000 and 8000.

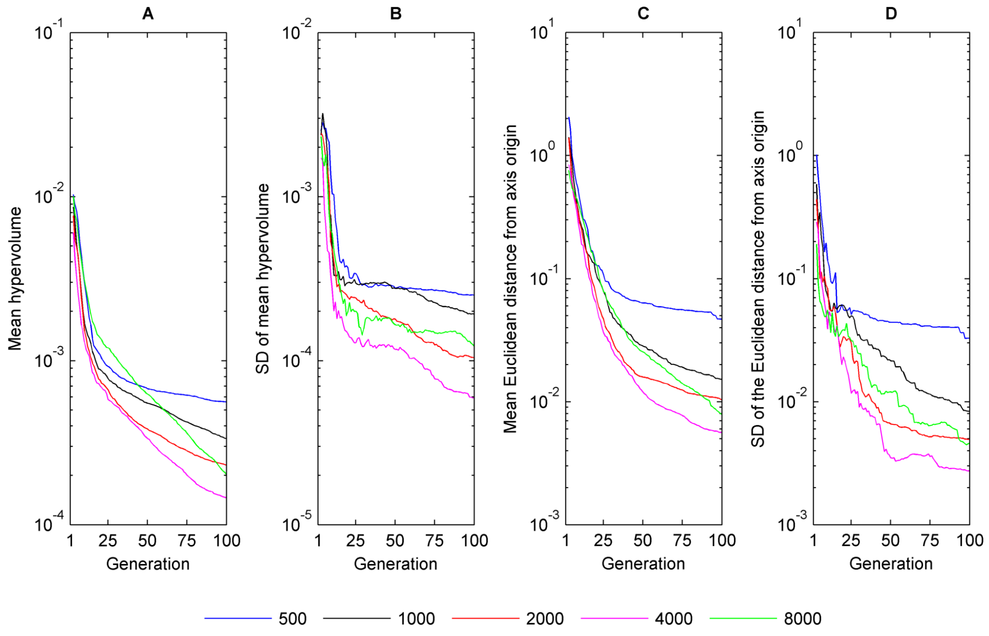

Figure S10: **Convergence metrics of NSGA-II when fitting the model to synthetic nystagmus waveform C of Fig. S4.** (A) Mean value of the hypervolume indicator $\mathcal{H}_I$ as a function of generation number n. (B) Standard deviation (SD) of $\mathcal{H}_I$ as a function of n. (C) Mean value of the smallest Euclidean distance $d_{\hat{\mathcal{F}}}$ between the Pareto front estimate and objective space origin as a function of n. (D) SD of $d_{\hat{\mathcal{F}}}$ as a function of n. Convergence metrics were calculated from 16 runs of NSGA-II each for the following population sizes: 500, 1000, 2000, 4000 and 8000.

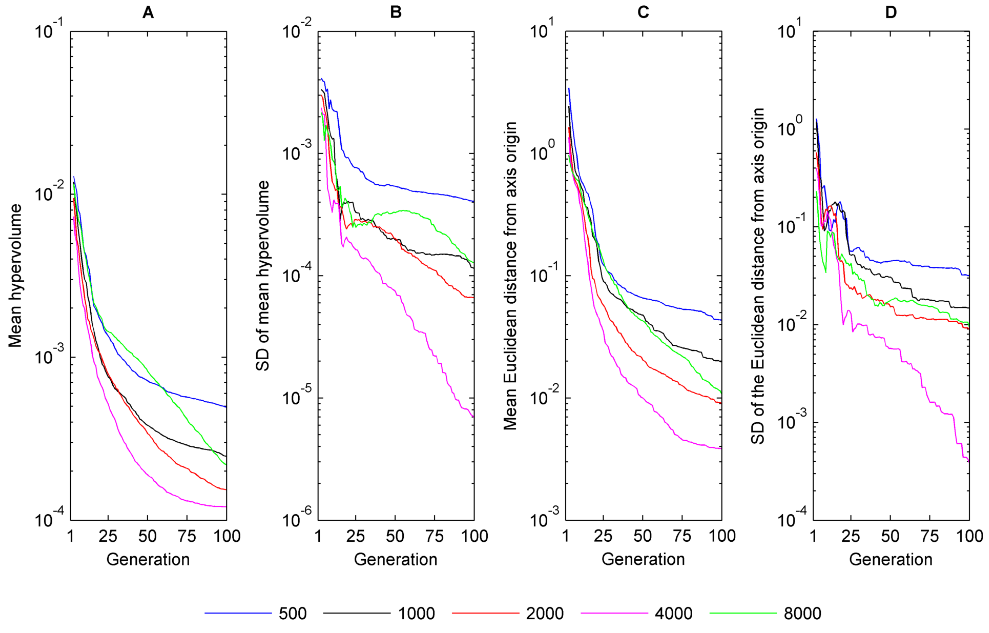

Figure S11: **Convergence metrics of NSGA-II when fitting the model to synthetic nystagmus waveform D of Fig. S4.** (A) Mean value of the hypervolume indicator $\mathcal{H}_I$ as a function of generation number n. (B) Standard deviation (SD) of $\mathcal{H}_I$ as a function of n. (C) Mean value of the smallest Euclidean distance $d_{\hat{\mathcal{F}}}$ between the Pareto front estimate and objective space origin as a function of n. (D) SD of $d_{\hat{\mathcal{F}}}$ as a function of n. Convergence metrics were calculated from 16 runs of NSGA-II each for the following population sizes: 500, 1000, 2000, 4000 and 8000.

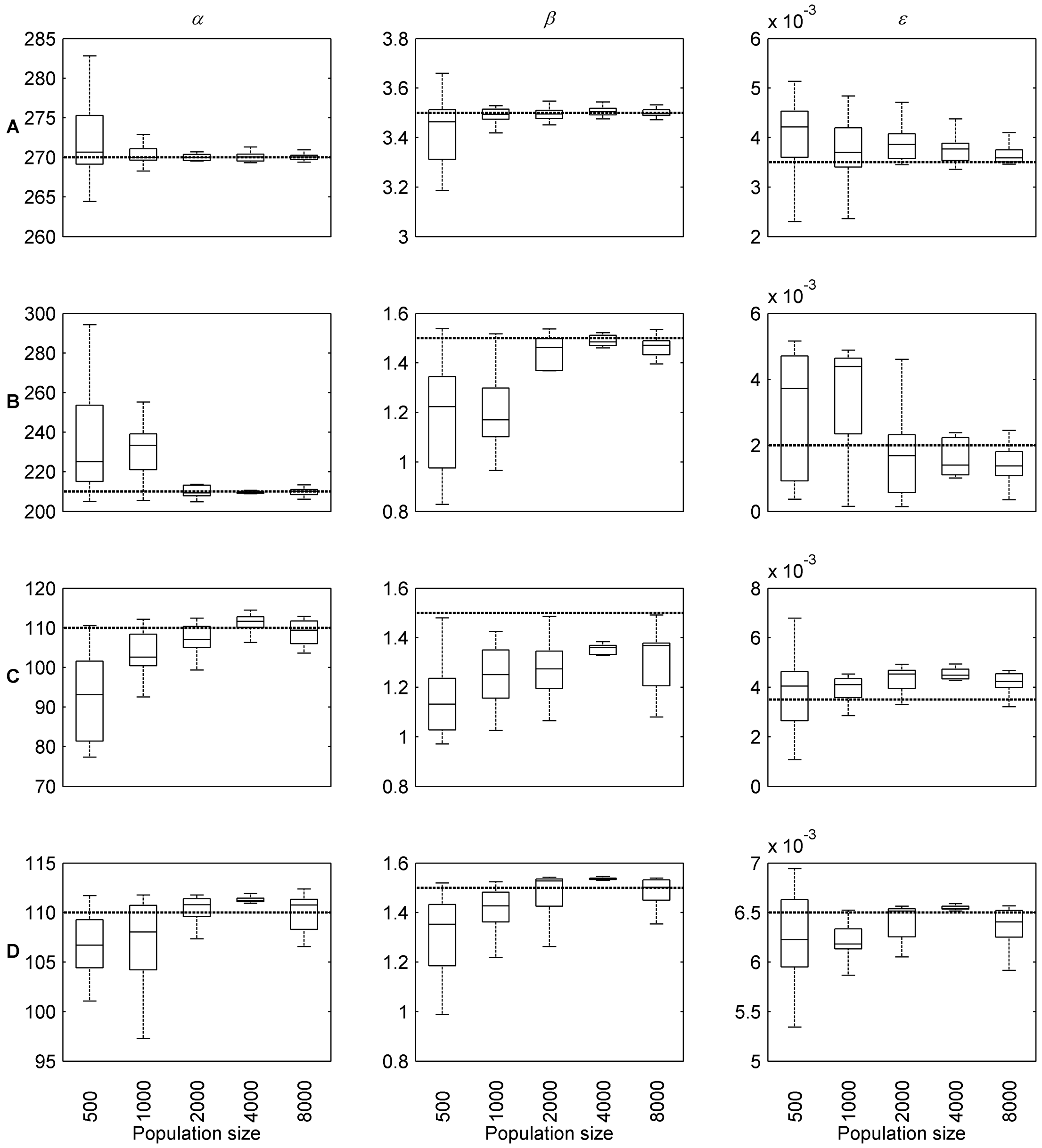

Figure S12: **Optimised parameter values for synthetic nystagmus waveforms as a function of population size.** The first line of plots shows the optimised values of α, β and ϵ for waveform A, whereas those for B, C and D are shown by the second, third and fourth lines of plots, respectively. In each plot, the dotted line represents the parameter value used to generate the target waveform. The horizontal line in each boxplot denotes the median of the optimised parameter values. The edges of each box are the 25th and 75th percentiles. The whiskers extend to the interquartile range.

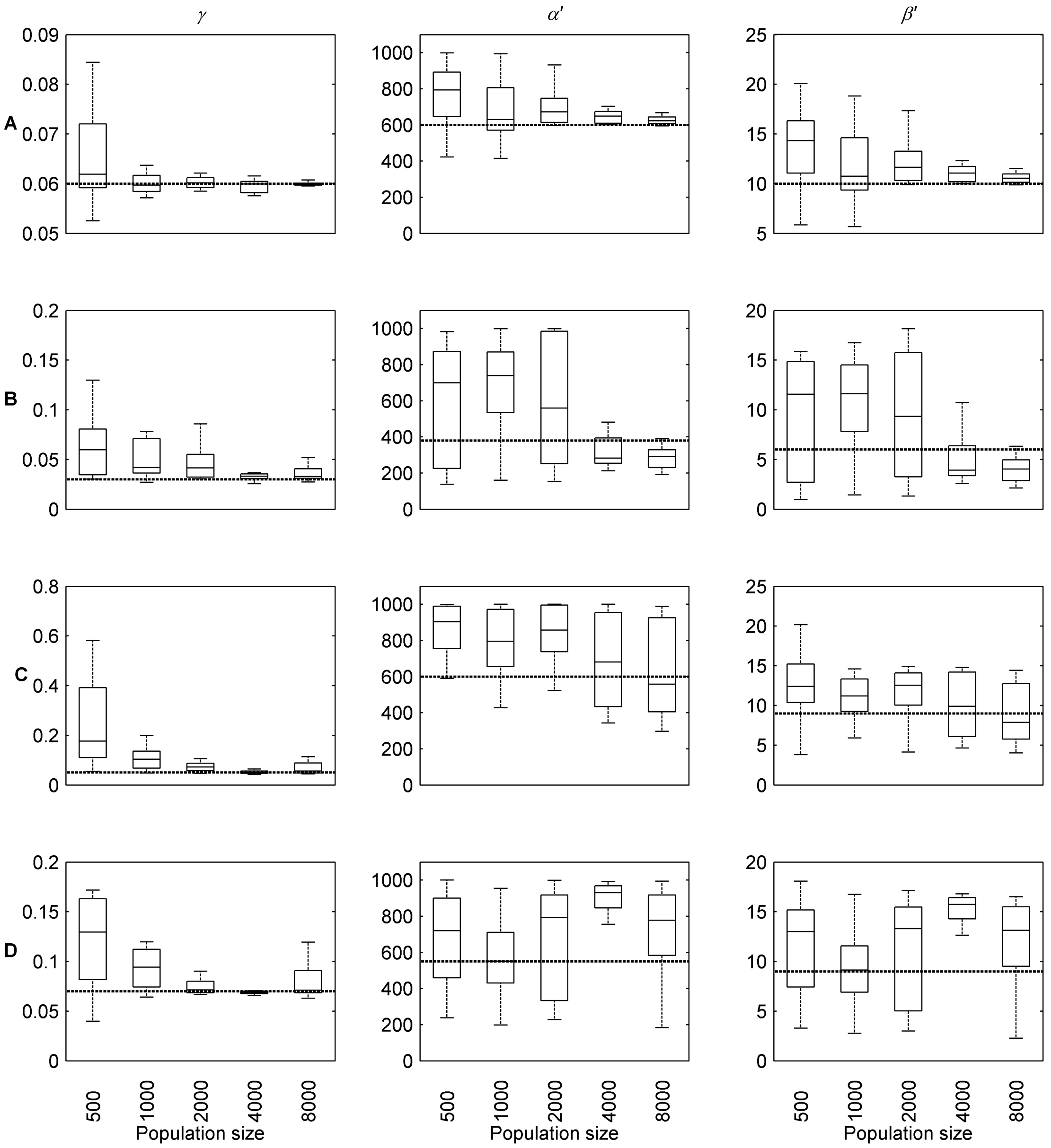

Figure S13: **Optimised parameter values for synthetic nystagmus waveforms as a function of population size.** The first line of plots shows the optimised values of γ, α' and β' for waveform A, whereas those for B, C and D are shown by the second, third and fourth lines of plots, respectively. In each plot, the dotted line represents the parameter value used to generate the target waveform. The horizontal line in each boxplot denotes the median of the optimised parameter values. The edges of each box are the 25th and 75th percentiles. The whiskers extend to the interquartile range.

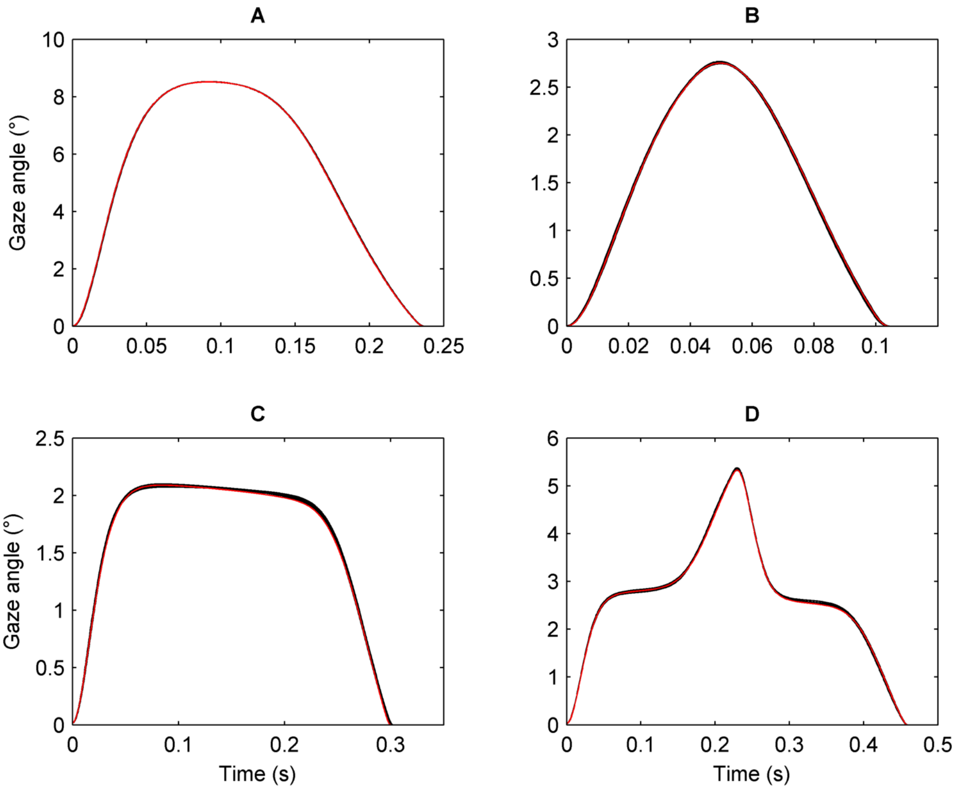

Figure S14: **Fits of the model to synthetic nystagmus waveforms.** The target waveforms are plotted in red (cf. Fig. S4). The optimal fits obtained from 16 independent runs of NSGA-II with a population size of 4000 are plotted in black. In each plot, the vertical axis represents the horizontal gaze angle in degrees (°), with positive values denoting rightward eye positions. Time is in seconds (s). The simulated nystagmus waveform types are: asymmetric pseudo-cycloid (A); pseudo-cycloid (B); jerk (C); bidirectional jerk (D).

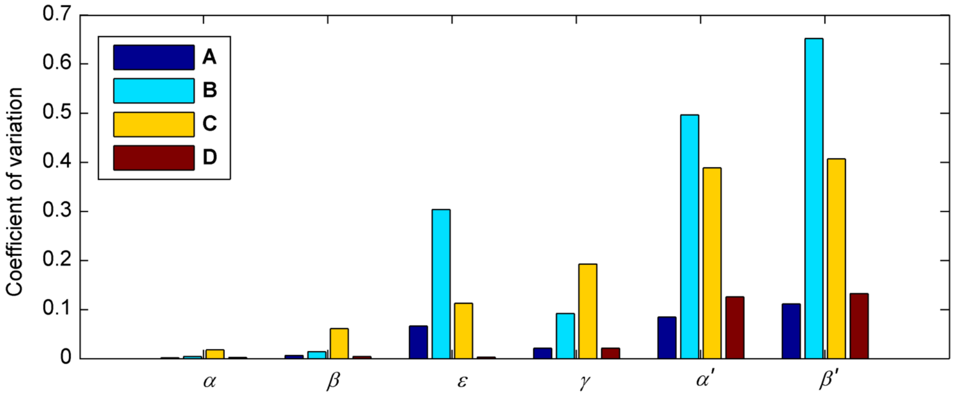

Figure S15: **Optimising the model to synthetic nystagmus waveforms: coefficients of variation of the parameters.** Coefficients of variation were calculated from 16 NSGA-II runs with a population size of 4000 each.

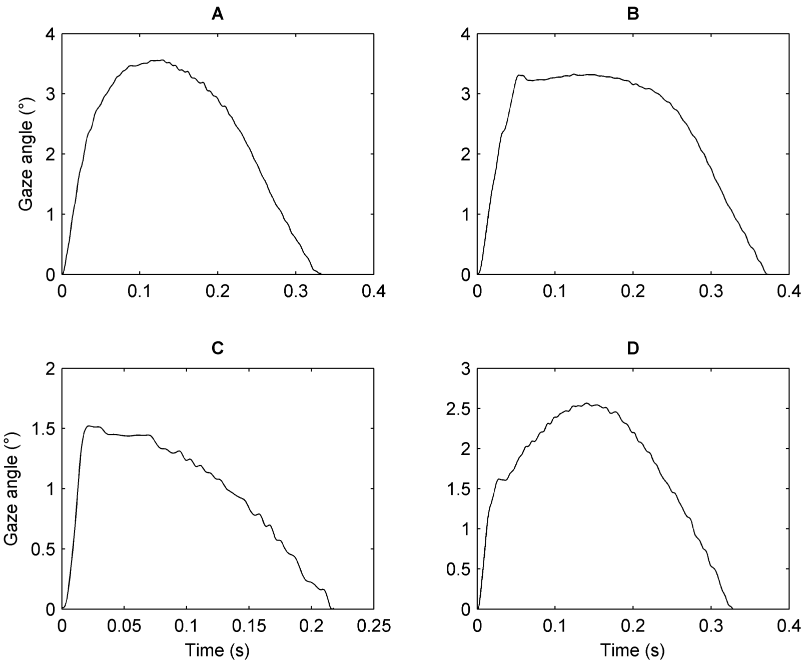

Figure S16: **Unstable periodic orbits (UPOs) extracted from the experimental time series shown in Fig. 4.** Each UPO was translated in time and linearly scaled so that it began at the start of the nystagmus fast phase, with the fast phase in the rightward direction. The oculomotor model was fitted to these normalised waveforms. On each plot, the vertical axis represents the horizontal gaze angle in degrees (°), with positive values denoting rightward eye positions. Time is in seconds (s). The nystagmus waveform types are: asymmetric pseudo-cycloid (A); jerk with extended foveation (B); jerk (C); and asymmetric pseudo-cycloid (D).

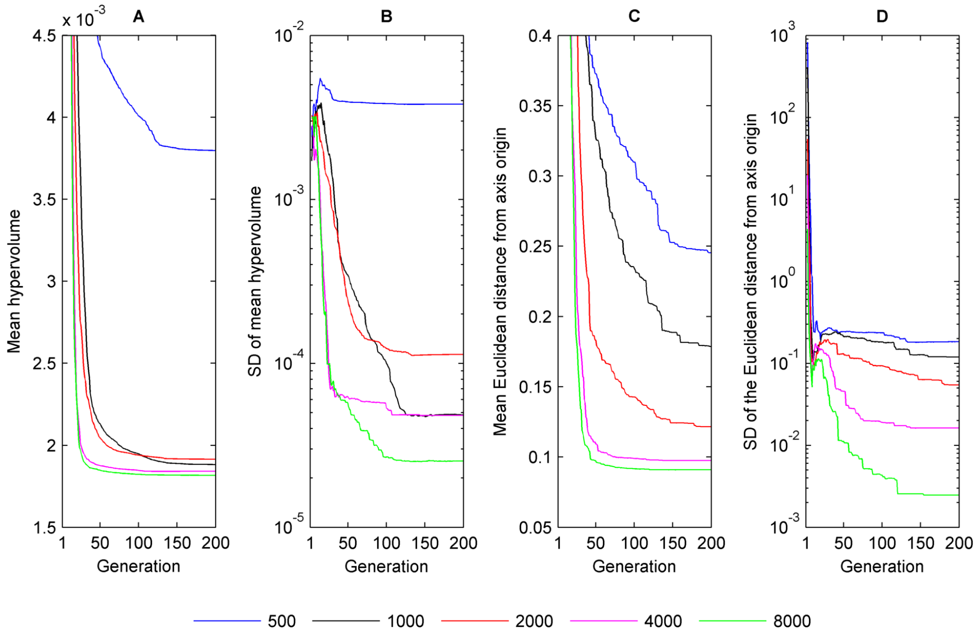

Figure S17: **Convergence metrics of NSGA-II when fitting the model to experimental nystagmus waveform B of Fig. 4.** (A) Mean value of the hypervolume indicator $\mathcal{H}_I$ as a function of generation number n. (B) Standard deviation (SD) of $\mathcal{H}_I$ as a function of n. (C) Mean value of the smallest Euclidean distance $d_{\hat{\mathcal{F}}}$ between the Pareto front estimate and objective space origin as a function of n. (D) SD of $d_{\hat{\mathcal{F}}}$ as a function of n. Convergence metrics were calculated from 16 runs of NSGA-II each for the following population sizes: 500, 1000, 2000, 4000 and 8000.

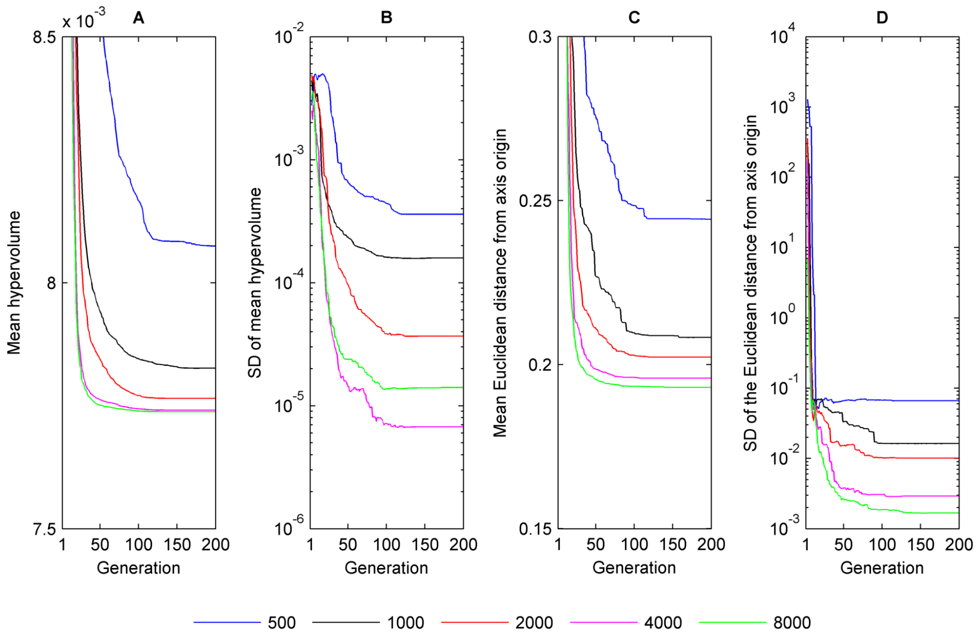

Figure S18: **Convergence metrics of NSGA-II when fitting the model to experimental nystagmus waveform C of Fig. 4.** (A) Mean value of the hypervolume indicator $\mathcal{H}_I$ as a function of generation number n. (B) Standard deviation (SD) of $\mathcal{H}_I$ as a function of n. (C) Mean value of the smallest Euclidean distance $d_{\hat{\mathcal{F}}}$ between the Pareto front estimate and objective space origin as a function of n. (D) SD of $d_{\hat{\mathcal{F}}}$ as a function of n. Convergence metrics were calculated from 16 runs of NSGA-II each for the following population sizes: 500, 1000, 2000, 4000 and 8000.

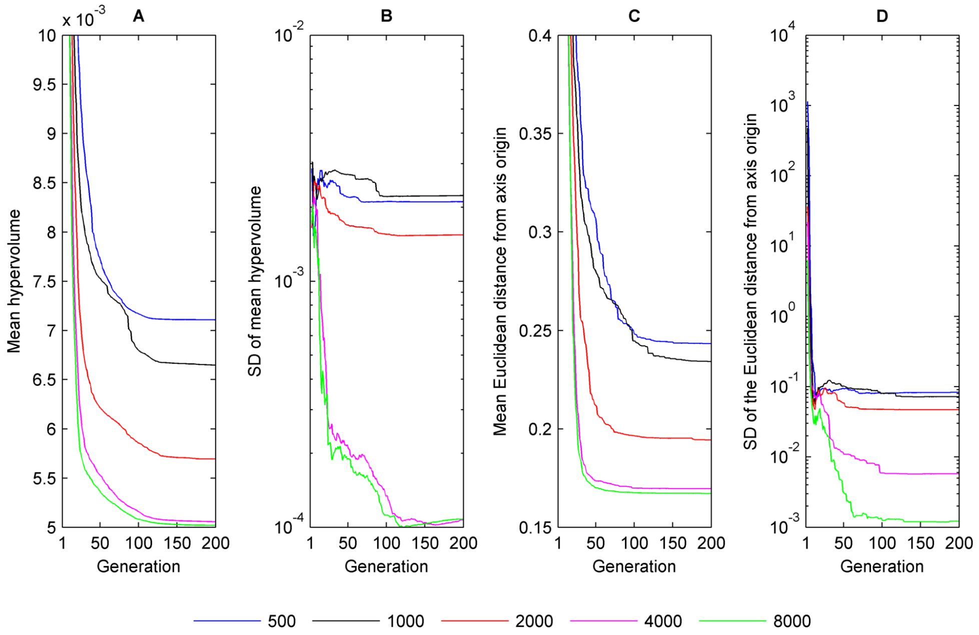

Figure S19: **Convergence metrics of NSGA-II when fitting the model to experimental nystagmus waveform D of Fig. 4.** (A) Mean value of the hypervolume indicator $\mathcal{H}_I$ as a function of generation number n. (B) Standard deviation (SD) of $\mathcal{H}_I$ as a function of n. (C) Mean value of the smallest Euclidean distance $d_{\hat{\mathcal{F}}}$ between the Pareto front estimate and objective space origin as a function of n. (D) SD of $d_{\hat{\mathcal{F}}}$ as a function of n. Convergence metrics were calculated from 16 runs of NSGA-II each for the following population sizes: 500, 1000, 2000, 4000 and 8000.

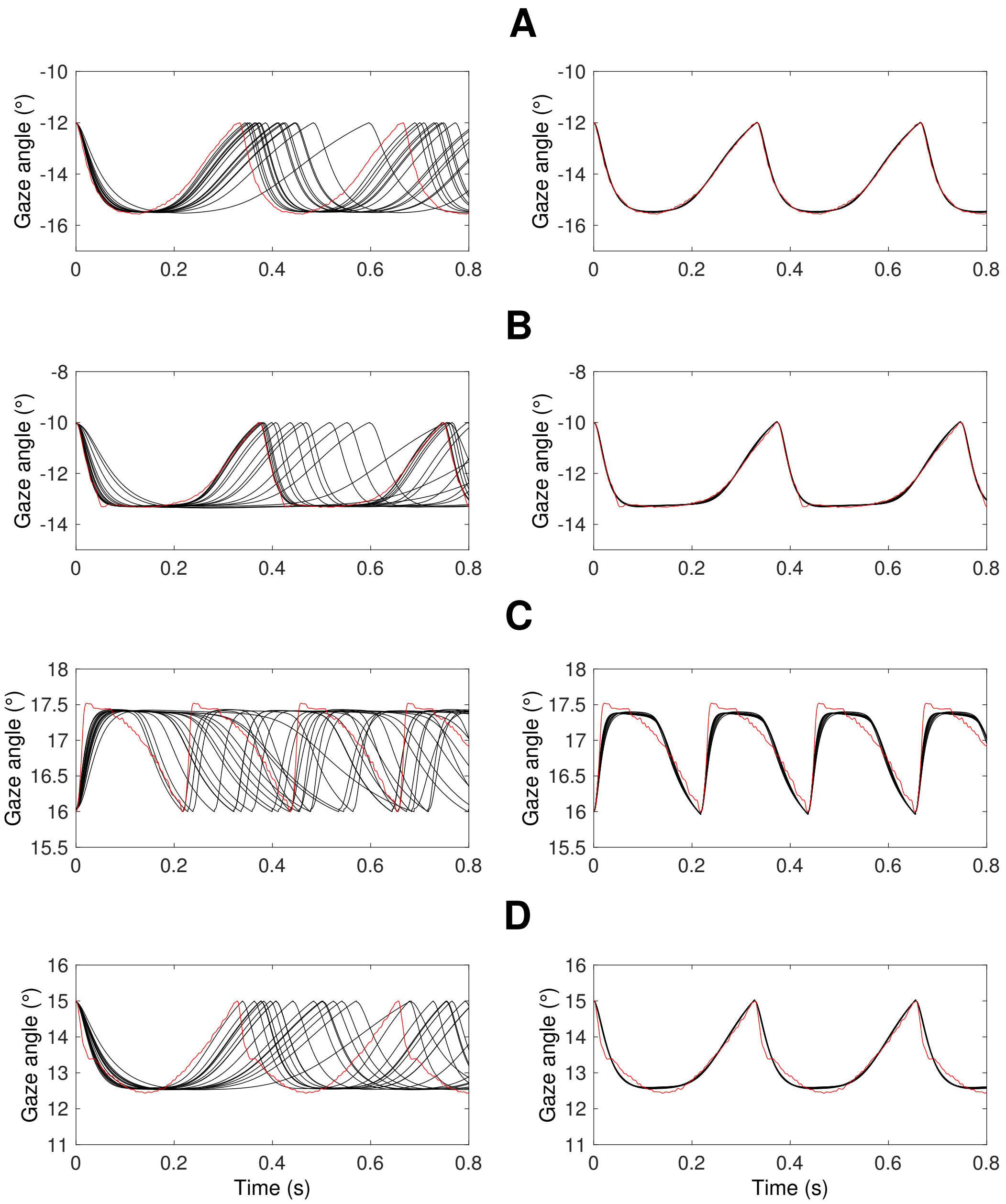

Figure S20: **Fits of the model to experimental nystagmus waveforms.** The target waveforms are plotted in red. Left panel: the optimised waveforms obtained from 16 independent runs of NSGA-II with a population size of 4000 (black lines). For each optimisation run, the final best-fit parameter set was selected as that yielding the minimum shape difference from the experimental oscillation. Right panel: the optimised waveforms scaled in time to match the period of the corresponding target waveform. In all plots, the vertical axis represents the horizontal gaze angle in degrees ($^{\circ}$), with positive values denoting rightward eye positions. Time is in seconds (s).

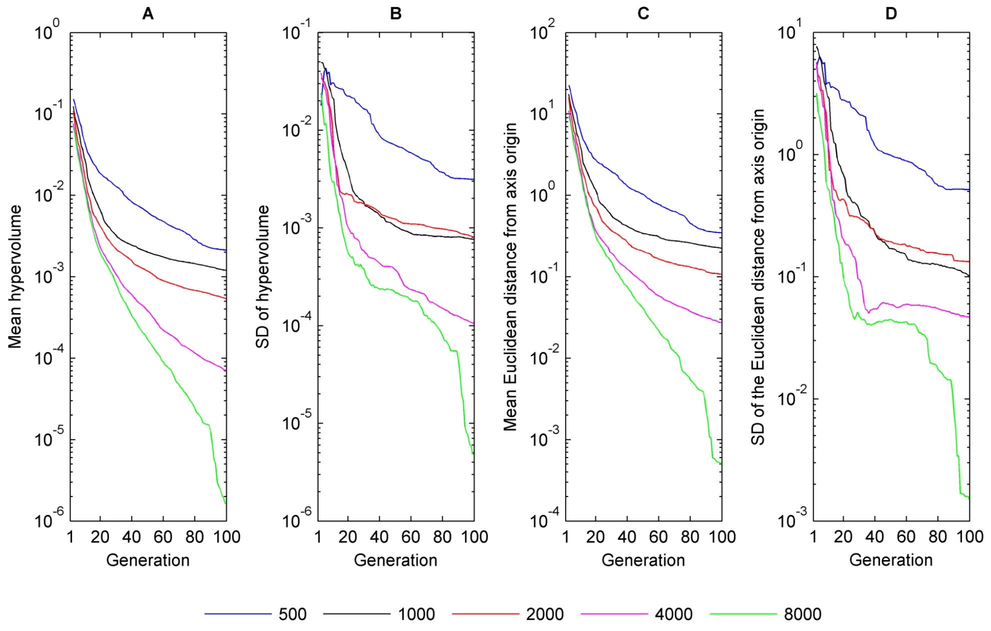

Figure S21: **Convergence metrics of NSGA-II when fitting the model to synthetic saccadic velocity profile A of Fig. S5.** (A) Mean value of the hypervolume indicator $\mathcal{H}_I$ as a function of generation number n. (B) Standard deviation (SD) of $\mathcal{H}_I$ as a function of n. (C) Mean value of the smallest Euclidean distance $d_{\hat{\mathcal{F}}}$ between the Pareto front estimate and objective space origin as a function of n. (D) SD of $d_{\hat{\mathcal{F}}}$ as a function of n. Convergence metrics were calculated from 16 runs of NSGA-II each for the following population sizes: 500, 1000, 2000, 4000 and 8000.

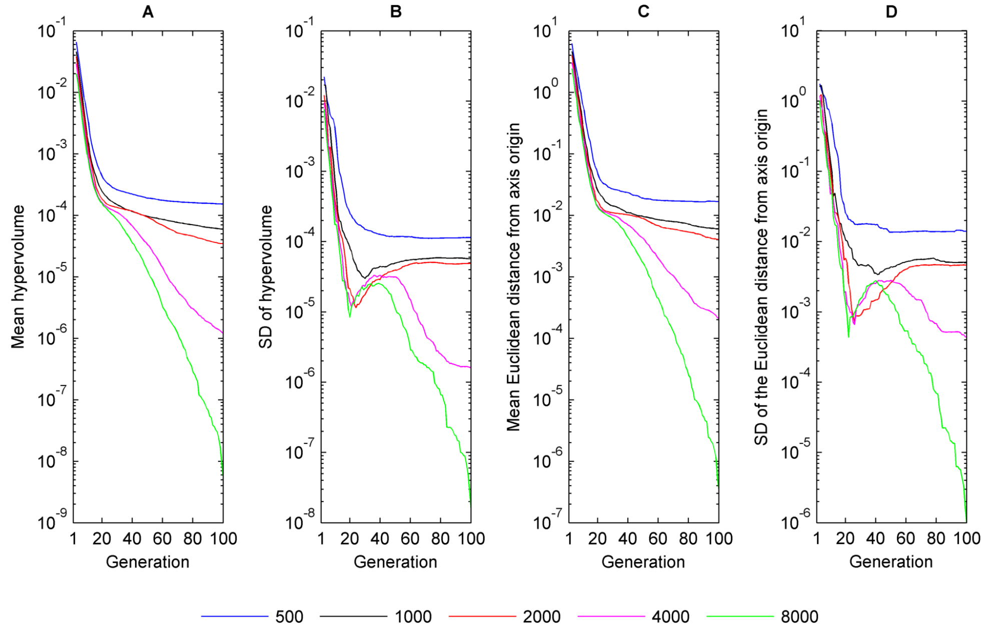

Figure S22: **Convergence metrics of NSGA-II when fitting the model to synthetic saccadic velocity profile B of Fig. S5.** (A) Mean value of the hypervolume indicator $\mathcal{H}_I$ as a function of generation number n. (B) Standard deviation (SD) of $\mathcal{H}_I$ as a function of n. (C) Mean value of the smallest Euclidean distance $d_{\hat{\mathcal{F}}}$ between the Pareto front estimate and objective space origin as a function of n. (D) SD of $d_{\hat{\mathcal{F}}}$ as a function of n. Convergence metrics were calculated from 16 runs of NSGA-II each for the following population sizes: 500, 1000, 2000, 4000 and 8000.

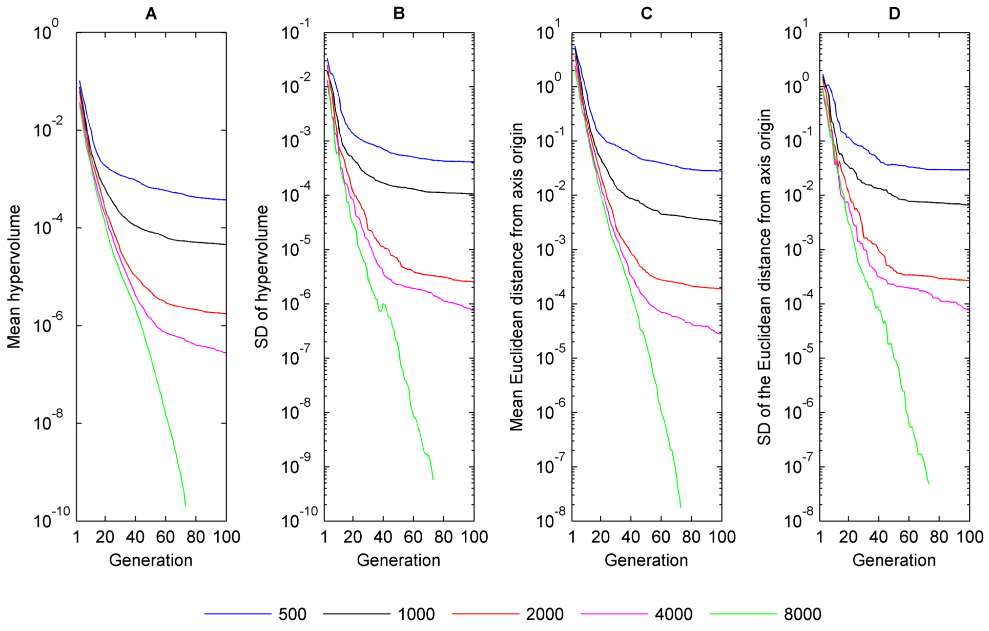

Figure S23: **Convergence metrics of NSGA-II when fitting the model to synthetic saccadic velocity profile C of Fig. S5.** (A) Mean value of the hypervolume indicator $\mathcal{H}_I$ as a function of generation number n. (B) Standard deviation (SD) of $\mathcal{H}_I$ as a function of n. (C) Mean value of the smallest Euclidean distance $d_{\hat{\mathcal{F}}}$ between the Pareto front estimate and objective space origin as a function of n. (D) SD of $d_{\hat{\mathcal{F}}}$ as a function of n. Convergence metrics were calculated from 16 runs of NSGA-II each for the following population sizes: 500, 1000, 2000, 4000 and 8000.

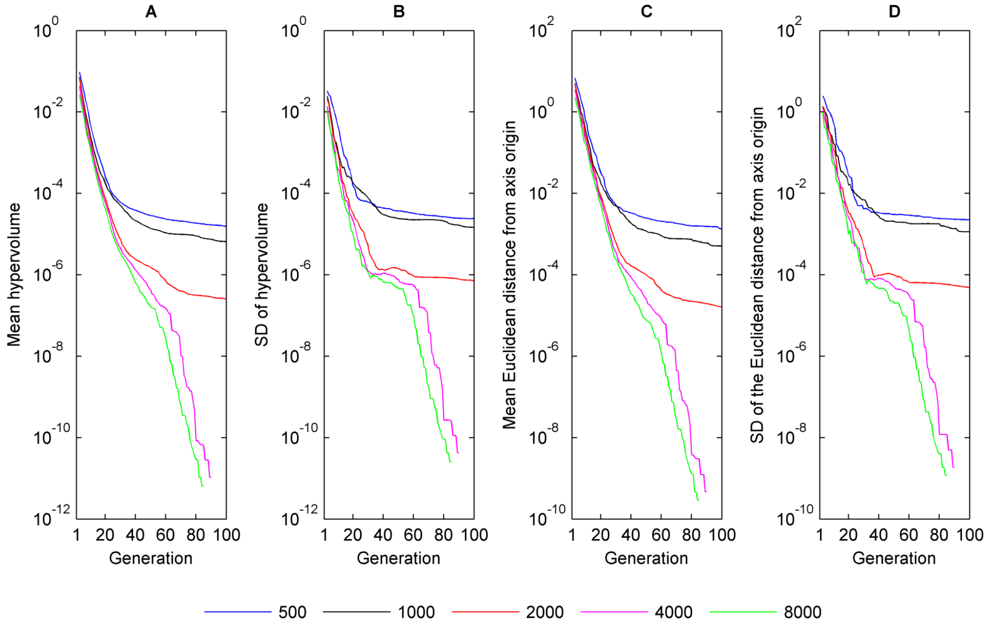

Figure S24: **Convergence metrics of NSGA-II when fitting the model to synthetic saccadic velocity profile D of Fig. S5.** (A) Mean value of the hypervolume indicator $\mathcal{H}_I$ as a function of generation number n. (B) Standard deviation (SD) of $\mathcal{H}_I$ as a function of n. (C) Mean value of the smallest Euclidean distance $d_{\hat{\mathcal{F}}}$ between the Pareto front estimate and objective space origin as a function of n. (D) SD of $d_{\hat{\mathcal{F}}}$ as a function of n. Convergence metrics were calculated from 16 runs of NSGA-II each for the following population sizes: 500, 1000, 2000, 4000 and 8000.

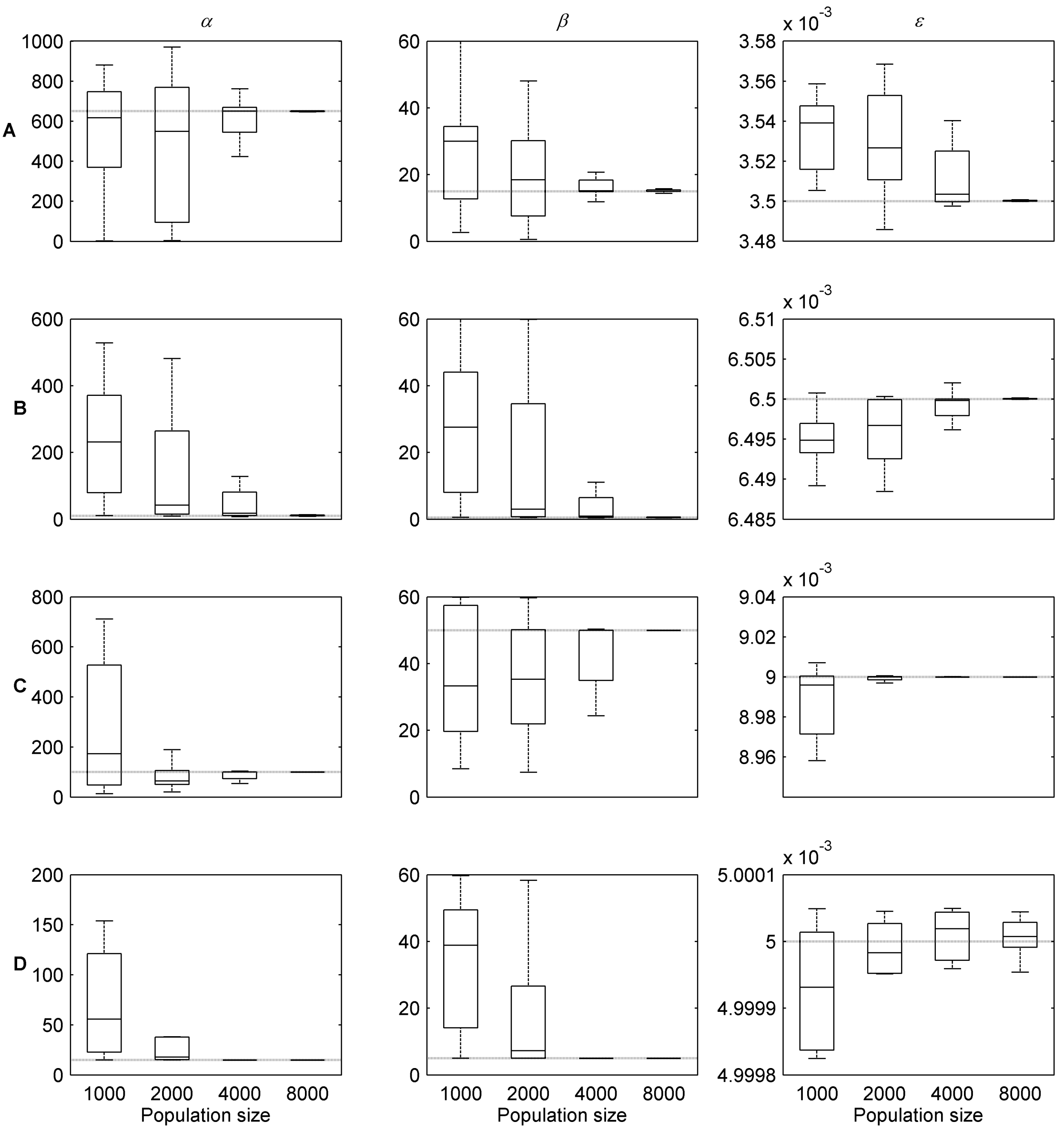

Figure S25: **Optimised parameter values for synthetic saccadic velocity profiles versus population size.** The first line of plots shows the optimised values of α, β and ϵ for velocity profile A of Fig. S5, whereas those for B, C and D are shown by the second, third and fourth lines of plots, respectively. In each plot, the dotted line represents the parameter value used to generate the target profile. The horizontal line in each boxplot denotes the median of the optimised parameter values. The edges of each box are the 25th and 75th percentiles. The whiskers extend to the outermost data points not considered as outliers.

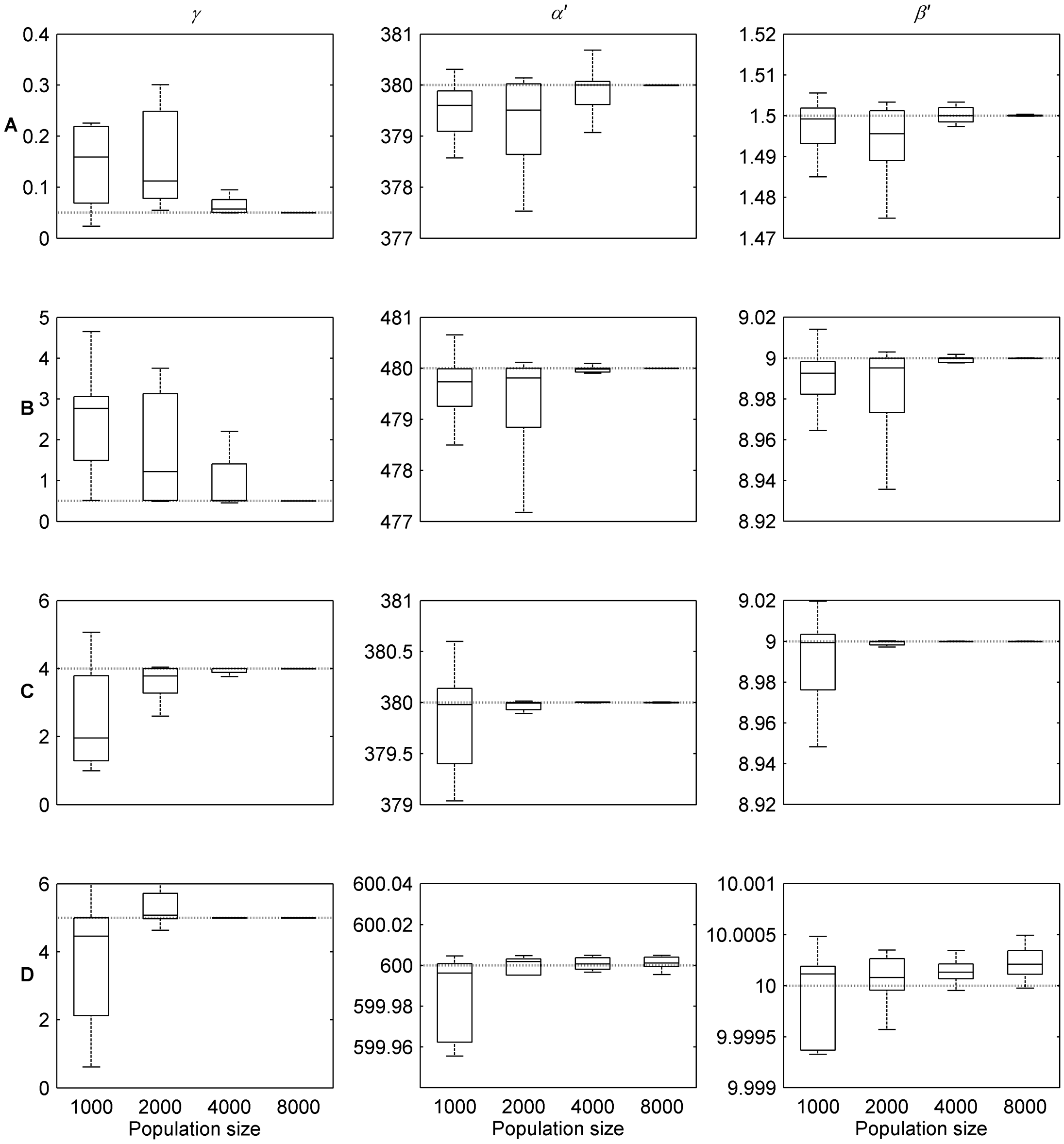

Figure S26: **Optimised parameter values for synthetic saccadic velocity profiles versus population size.** The first line of plots shows the optimised values of γ, α' and β' for velocity profile A of Fig. S5, whereas those for B, C and D are shown by the second, third and fourth lines of plots, respectively. In each plot, the dotted line represents the parameter value used to generate the target profile. The black horizontal line in each boxplot denotes the median of the optimised parameter values. The edges of each box are the 25th and 75th percentiles. The whiskers extend to the outermost data points not considered as outliers.

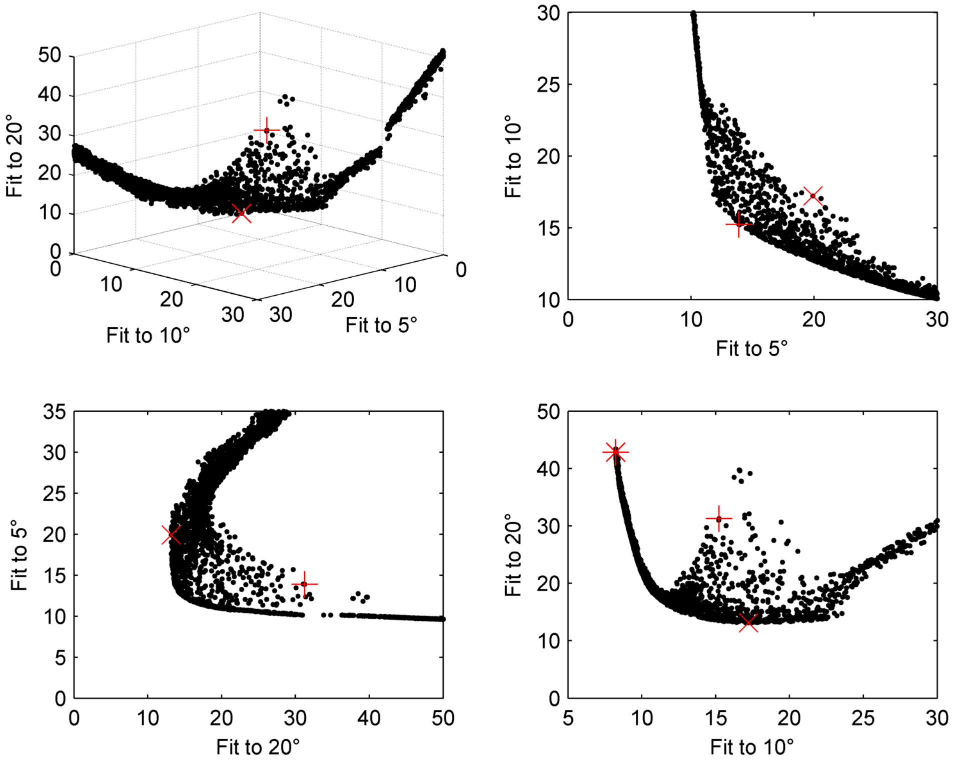

Figure S27: **Magnified views of the Pareto front shown in Fig. 13 about the origin of objective space**. In each plot, the red cross (+), red star (∗) and red x (×) indicate the solutions yielding the minimum Euclidean distance to the axes origin, the best fit to a 10 degree saccade and the best fit to a 20 degree saccade, respectively.

Supplementary Tables

	α		β		ϵ		γ		α'		β'	
**Target**	**M**	**O**	**M**	**O**	**M**	**O**	**M**	**O**	**M**	**O**	**M**	**O**
A	270	270.0600 (0.0029)	3.5	3.5034 (0.0070)	0.0035	0.0036 (0.0703)	0.06	0.0597 (0.0201)	600	630.5900 (0.0846)	10	10.6700 (0.1134)
B	210	210.4200 (0.0046)	1.5	1.4612 (0.0144)	0.0020	0.0015 (0.3360)	0.03	0.0358 (0.0922)	380	340.8200 (0.4961)	6	5.0800 (0.6514)
C	110	109.1300 (0.0157)	1.5	1.3136 (0.0625)	0.0035	0.0042 (0.0970)	0.05	0.0650 (0.1753)	600	623.0900 (0.3914)	9	8.8200 (0.4111)
D	110	109.8000 (0.0025)	1.5	1.4781 (0.0047)	0.0065	0.0064 (0.0034)	0.07	0.0836 (0.0215)	550	723.3200 (0.1260)	9	11.9800 (0.1326)

Table S1: **Model parameter values (M) used to generate the synthetic nystagmus waveforms shown in Fig. S4, together with the parameter values obtained by optimising the model to the waveforms (O).** Mean optimised parameter values and coefficients of variation (shown in brackets) were calculated from 16 NSGA-II runs with a population size of 4000.

	α		β		ϵ		γ		α'		β'	
**Target**	**M**	**O**	**M**	**O**	**M**	**O**	**M**	**O**	**M**	**O**	**M**	**O**
A	650	643.7700 (0.0892)	15	15.3410 (0.2240)	0.0035	0.0035 (0.0025)	0.05	0.0530 (0.2078)	380	380.0200 (0.0003)	1.5	1.5000 (0.0008)
B	10	11.5900 (0.3279)	0.5	0.5844 (0.3366)	0.0065	0.0065 (2.9000e-5)	0.5	0.5031 (0.0173)	480	479.9900 (1.1226e-5)	9	8.9900 (1.4284e-5)
C	100	96.4400 (0.0791)	50	47.7500 (0.0929)	0.0090	0.0090 (2.9652e-6)	4	3.9780 (0.0069)	380	379.9900 (7.4419e-6)	9	8.9900 (3.1372e-6)
D	15	14.9900 (2.1836e-5)	5	4.9900 (6.1320e-6)	0.0050	0.0050 (5.1906e-6)	5	5 (6.4711e-6)	600	600 (4.8757e-6)	10	10 (1.7334e-5)

Table S2: **Model parameter values (M) used to generate the synthetic saccadic velocity profiles shown in Fig. S5, together with the parameter values obtained by optimising the model to the profiles (O).** Mean optimised parameter values and coefficients of variation (shown in brackets) were calculated from 16 NSGA-II runs with a population size of 8000.

**Population size**	**Computation time (h)**
500	4.04
1000	6.74
2000	14.54
4000	29.07
8000	58.22

Table S3: **Time required to run NSGA-II 16 times for the four synthetic nystagmus waveforms shown in Fig. S4 as a function of population size.** The computation times shown here were obtained using the multiple parallel runs method. The CPU used was the Intel i7-4790K and the GPU used was the AMD Firepro W8100.

α	β	ϵ	γ	α'	β'
73.8860	1.3798	0.000422	3.9058	941.9604	18.2249
74.0231	1.3574	0.000420	4.3308	959.7598	18.1994
74.1901	1.3626	0.000387	4.3381	936.7846	17.7744
74.0151	1.3949	0.000914	3.2675	976.9992	19.6056
74.3313	1.3609	0.000858	3.7233	752.4665	14.5887
73.4383	1.4080	0.000327	3.5781	986.3955	19.5748
73.1678	1.3670	0.000369	4.1287	693.3085	13.4952
74.2034	1.4041	0.001035	3.0163	932.5658	18.8773
73.9068	1.3890	0.000822	3.3548	983.6076	19.5408
73.8722	1.3604	0.000210	4.7199	878.9018	16.6145
74.0002	1.3630	0.000245	4.4958	871.5667	16.4806
74.4777	1.3433	0.000320	4.7195	938.8144	17.3741
74.4609	1.3621	0.000621	3.9958	965.4139	18.4023
73.6851	1.3846	0.000303	4.0965	905.3063	17.5976
74.6357	1.3626	0.000378	4.5059	984.3670	18.5614
73.9528	1.3787	0.000697	3.5725	899.9799	17.6012

Table S4: **Optimised parameter values for experimental nystagmus waveform A.** The parameter values were generated from 16 NSGA-II runs with a population size of 4000.

α	β	ϵ	γ	α'	β'
81.4470	1.2587	0.003587	2.6216	946.6078	15.7112
82.9527	1.1921	0.003561	3.7606	921.8840	14.2736
82.3008	1.2258	0.003570	3.0617	901.0864	14.4253
81.9359	1.2455	0.003619	2.7211	928.2800	15.1769
80.3064	1.2444	0.002190	3.7432	960.2521	15.3978
81.7844	1.2508	0.003273	2.8519	987.0697	16.0224
82.4753	1.2332	0.003513	2.9892	984.6942	15.7731
81.8098	1.2367	0.003029	3.2038	971.0881	15.4672
82.3850	1.1939	0.004027	3.0283	662.3819	10.6367
82.6411	1.2254	0.003366	3.1833	961.3344	15.1860
81.8225	1.2454	0.003443	2.8889	950.9924	15.4640
80.9086	1.2430	0.003157	3.1219	988.4403	16.0739
82.8045	1.2179	0.003388	3.3524	995.3751	15.6161
84.2661	1.1924	0.003573	3.2569	762.1264	11.6122
82.8931	1.2432	0.003954	2.6408	962.7046	15.8182
82.8692	1.2122	0.003382	3.3613	995.3670	15.5252

Table S5: **Optimised parameter values for experimental nystagmus waveform B.** The parameter values were generated from 16 NSGA-II runs with a population size of 4000.

α	β	ϵ	γ	α'	β'
63.8105	0.3796	0.001603	149.3738	513.0984	3.3434
58.7743	0.3982	0.001108	130.5800	843.9449	5.9600
59.5450	0.4076	0.001589	104.4812	771.5557	5.7317
61.8556	0.4067	0.001119	145.6253	934.5026	6.4123
60.3481	0.3987	0.001333	146.5753	749.3091	5.2715
60.0300	0.3930	0.001559	146.0221	946.6963	6.7319
56.4527	0.3997	0.001378	139.7951	999.4586	7.6674
60.3975	0.4059	0.001623	146.1372	965.0309	7.1929
58.0912	0.4032	0.001262	140.1972	971.2520	7.1726
58.3580	0.3928	0.001519	132.9635	738.6576	5.3861
59.2070	0.4108	0.001166	146.9515	968.8204	7.1303
65.6467	0.3808	0.001436	135.1793	323.1411	2.0052
60.2424	0.4018	0.001477	145.1551	743.2897	5.3772
61.6694	0.3977	0.001362	135.5100	943.4591	6.4358
58.9473	0.4041	0.001627	131.8488	757.3143	5.7652
60.6373	0.3935	0.001213	148.0121	877.3826	5.9659

Table S6: **Optimised parameter values for experimental nystagmus waveform C.** The parameter values were generated from 16 NSGA-II runs with a population size of 4000.

α	β	ϵ	γ	α'	β'
59.1972	1.1058	0.000607	2.5306	970.0289	18.6077
58.1421	1.1364	0.000551	2.2883	991.9926	19.9460
57.7539	1.1768	0.000226	2.1399	930.6437	19.4500
57.1412	1.1863	0.000311	2.0269	961.1829	20.5195
58.9697	1.1387	0.000581	2.1330	899.8404	17.8734
57.7300	1.1500	0.000417	2.1764	720.9010	14.7580
58.9381	1.2071	0.000650	1.5444	940.3915	19.9185
58.2983	1.1283	0.000468	2.3509	958.6452	19.0491
58.2391	1.1803	0.000779	1.7230	924.6075	19.4091
57.8602	1.1942	0.000965	1.5643	949.3527	20.4036
60.7869	0.9883	0.000234	4.7916	907.0333	14.9787
58.4281	1.1596	0.000403	2.1045	909.8404	18.5493
57.6040	1.1671	0.000733	2.0505	975.9734	20.4760
60.4556	1.1088	0.000553	2.3623	958.9046	18.0338
60.5561	1.0795	0.000630	2.6750	760.5867	13.8953
58.9817	1.1149	0.000283	2.5673	959.4191	18.5497

Table S7: **Optimised parameter values for experimental nystagmus waveform D.** The parameter values were generated from 16 NSGA-II runs with a population size of 4000.

α	β	ϵ	γ	α'	β'
5.2979	18.1755	0.007484	3.5454×10^{-5}	536.3145	3.3027
17.1781	54.9156	0.007377	4.5276×10^{-5}	539.8244	3.3820
31.5053	21.5546	0.007419	1.0772×10^{-5}	543.4726	3.3798
6.4617	30.2693	0.007482	4.2245×10^{-5}	539.3890	3.3385
25.2468	46.8037	0.007535	3.6671×10^{-5}	541.0604	3.3839
22.8235	54.2920	0.007432	2.0201×10^{-5}	536.8340	3.3265
7.2052	35.6141	0.007323	7.3297×10^{-5}	525.5349	3.2087
33.2020	57.2672	0.007356	1.6723×10^{-5}	534.6402	3.3163
24.6312	46.3618	0.007409	2.7822×10^{-5}	546.5006	3.4964
9.9587	3.6352	0.007317	2.5811×10^{-5}	528.8543	3.2369
4.6892	49.8071	0.007371	2.3472×10^{-5}	537.8095	3.3573
25.8110	33.8400	0.007350	4.3886×10^{-5}	534.7941	3.2588
4.5098	38.8240	0.007488	1.7915×10^{-5}	543.1064	3.4270
17.6225	56.5199	0.007445	4.3512×10^{-5}	536.9375	3.3293
7.4677	20.2686	0.007518	5.2804×10^{-5}	546.8587	3.4265
19.7280	11.0263	0.007359	3.0950×10^{-5}	530.4772	3.2473

Table S8: **Optimised parameter values for experimental saccades with selection method A.** The parameter values were generated from 16 NSGA-II runs with a population size of 8000.

α	β	ϵ	γ	α'	β'
107.8408	0.6870	0.003887	0.0106	251.2221	0.4583
41.0974	0.6087	0.003822	0.0846	249.0326	0.3955
553.2830	16.8743	0.003707	0.1074	248.2133	0.3650
139.7792	4.0468	0.003822	0.0888	249.3472	0.3774
281.3422	9.0625	0.003835	0.0875	249.2597	0.3785
79.4163	2.2230	0.003823	0.0988	248.9210	0.3755
111.8899	2.1975	0.003901	0.0440	250.8227	0.4135
555.1691	12.8995	0.003646	0.0710	247.5061	0.3617
71.9387	0.4238	0.003822	0.0262	248.7433	0.4101
331.8248	13.2771	0.003789	0.1360	248.5950	0.3728
104.6945	0.1150	0.004025	0.0616	251.9270	0.4961
72.8745	1.6161	0.003827	0.0720	248.9827	0.3887
20.6609	0.3466	0.003923	0.1861	250.5856	0.3986
737.2948	32.5337	0.003728	0.1792	247.2789	0.3554
480.5098	13.7527	0.003751	0.0845	248.5587	0.3805
540.9923	11.7577	0.003675	0.0593	248.3142	0.3715

Table S9: **Optimised parameter values for experimental saccades with selection method B.** The parameter values were generated from 16 NSGA-II runs with a population size of 8000.

α	β	ϵ	γ	α'	β'
117.4972	16.8053	0.004472	0.019742	396.6031	1.0778
62.0893	38.4591	0.005657	0.000956	432.1898	1.6798
43.3139	52.6766	0.004981	0.004977	410.4475	1.3443
625.6596	47.1506	0.004595	0.009403	399.1188	1.1028
270.3753	50.0047	0.005243	0.002635	422.2609	1.5114
20.9223	23.0898	0.004602	0.018887	399.6315	1.1164
346.6928	44.2214	0.004873	0.007016	408.5792	1.2680
100.7620	38.0061	0.005480	0.001103	431.4354	1.7658
27.6615	13.6133	0.005283	0.002652	421.2316	1.5262
168.7763	22.1677	0.004296	0.039950	393.3885	1.0756
188.8032	48.9628	0.005307	0.002097	423.2731	1.6164
281.0069	29.0410	0.004841	0.005568	404.9369	1.2435
52.1547	57.0190	0.004372	0.031414	392.6982	1.0516
55.8875	45.7220	0.004493	0.036287	402.8819	1.3013
49.8047	34.3625	0.004713	0.012601	402.6626	1.1754
202.5608	32.0198	0.005001	0.005011	411.1283	1.2969

Table S10: **Optimised parameter values for experimental saccades with selection method C.** The parameter values were generated from 16 NSGA-II runs with a population size of 8000.

α	β	ϵ	γ	α'	β'
74.2874	20.9476	0.005597	0.000739	466.8249	2.8925
63.6664	58.2268	0.005672	0.000776	468.8691	2.9175
6.4792	49.0435	0.005728	0.000542	468.9678	2.9665
12.7385	37.7213	0.005640	0.001045	467.8222	2.9300
50.0420	47.2101	0.005667	0.000797	468.5883	2.9632
22.7009	32.2828	0.005653	0.000575	467.6818	2.9474
8.6139	31.2337	0.005739	0.000844	469.6473	2.9531
37.4401	42.0355	0.005691	0.000549	468.7901	2.9655
1.1778	6.1920	0.005687	0.000632	468.5299	2.9479
86.3693	44.7407	0.005626	0.000750	467.9718	2.9251
21.2250	47.9045	0.005681	0.000894	468.6346	2.9330
6.9513	32.3392	0.005660	0.000773	468.1029	2.9334
15.4132	15.7937	0.005748	0.000674	469.5543	2.9361
74.4638	44.9056	0.005742	0.000995	470.8274	2.9909
64.9811	58.8444	0.005647	0.000745	467.4807	2.9264
131.1230	59.2050	0.005666	0.000571	468.6921	2.9494

Table S11: **Optimised parameter values for experimental saccades with selection method D.** The parameter values were generated from 16 NSGA-II runs with a population size of 8000.

References

[1] Ryoo S, Rodrigues CI, Baghsorkhi SS, Stone SS, Kirk DB, Hwu WmW: **Optimization principles and application performance evaluation of a multithreaded GPU using CUDA**. In *Proceedings of the 13th Symposium on Principles and Practice of Parallel Programming*, ACM 2008:73–82.

[2] Stone JE, Gohara D, Shi G: **OpenCL: a parallel programming standard for heterogeneous computing systems**. *Comput Sci Eng* 2010, **12**(1-3):66–73.

[3] Khronos Group: *The OpenCL Specification* 2010.

[4] Hairer E, Wanner G: *Solving Ordinary Differential Equations II.* Springer-Verlag 1991.

[5] Pasquariello G, Cesarelli M, Bifulco P, Fratini A, La Gatta A, Romano M: **Characterisation of baseline oscillation in congenital nystagmus eye movement recordings**. *Biomed Signal Process* 2009, **4**(2):102–107.

[6] Dell'Osso L, Daroff R: **Congenital nystagmus waveforms and foveation strategy**. *Doc Ophthalmol* 1975, **39**:155–182.
